# Metal-Free Addition/Head-to-Tail Polymerization of Transient Phosphinoboranes, RPH-BH_2_: A Route to Poly(alkylphosphinoboranes)

**DOI:** 10.1002/anie.201507084

**Published:** 2015-10-02

**Authors:** Christian Marquardt, Titel Jurca, Karl-Christian Schwan, Andreas Stauber, Alexander V Virovets, George R Whittell, Ian Manners, Manfred Scheer

**Affiliations:** Universität Regensburg, Institut für Anorganische Chemie 93040 Regensburg (Germany) E-mail: manfred.scheer@ur.de; School of Chemistry, Bristol University Cantock's Close, BS8 1TS, Bristol (UK) E-mail: ian.manners@bristol.ac.uk; Nikolaev Institute of Inorganic Chemistry SB RAS Lavrentiev str. 3, Novosibirsk 630090 (Russia) Novosibirsk State University Pirogova str. 2, Novosibirsk 630090 (Russia)

**Keywords:** addition polymerization, inorganic polymers, phosphinoboranes, phosphine–borane adducts, poly(phosphinoboranes)

## Abstract

Mild thermolysis of Lewis base stabilized phosphinoborane monomers R^1^R^2^P–BH_2_⋅NMe_3_ (R^1^,R^2^=H, Ph, or *t*Bu/H) at room temperature to 100 °C provides a convenient new route to oligo- and polyphosphinoboranes [R^1^R^2^P-BH_2_]_n_. The polymerization appears to proceed via the addition/head-to-tail polymerization of short-lived free phosphinoborane monomers, R^1^R^2^P-BH_2_. This method offers access to high molar mass materials, as exemplified by poly(*tert*-butylphosphinoborane), that are currently inaccessible using other routes (e.g. catalytic dehydrocoupling).

Polymers based on main-group elements other than carbon represent attractive materials as a result of their uses as elastomers, lithographic resists, biomaterials, polyelectrolytes, ceramic precursors, and in optoelectronics.[1, 2] Current routes to main-group-element macromolecules generally involve either polycondensation or ring-opening polymerization pathways. Metal-catalyzed polycondensation processes, such as cross-coupling and dehydrocoupling, have also attracted recent attention.[1p, 3] In contrast to the situation with organic polymer synthesis, the use of addition polymerization methods is rare, partly due to challenges associated with the generation of suitable multiply bonded monomers. Nevertheless, Gates and co-workers have shown that kinetically stable phosphaalkenes (MesP=C(Ar)Ph; Ar=Ph, C_6_H_4_OMe) undergo an addition–rearrangement polymerization in the presence of radical or anionic initiators.[1q, 4] Furthermore, Baines and co-workers have utilized anion-initiated addition polymerization of germenes and silenes (Mes_2_E=CHCH_2_*t*Bu; E=Ge, Si) to form polygermenes and poly(silylenemethylenes), respectively,[5] demonstrating the use of addition polymerization as a promising approach for the synthesis of main-group-element polymers.[6, 7]

Compounds with bonds between elements of Groups 13 and 15 are formally isoelectronic to their carbon analogues. However, due to electronegativity differences, the bonds are polar and lead to different physical and chemical properties.[8–10] The analogy has nevertheless stimulated the synthesis of a range of new molecules and materials such as BN analogues of pyrene,[11] carbon nanotubes,[12] and fullerene-like BN hollow spheres.[13] Counterparts of organic macromolecules have also attracted much attention and polymers based on poly(*p*-phenylene)-like cyclolinear structures involving borazines (polyborazylenes) have been studied in detail and, more recently, analogues of polyolefins, polyaminoboranes [RNH-BH_2_]_*n*_, have been isolated.[14]

Poly(phosphinoboranes) [RPH-BH_2_]_*n*_ have been prepared over the past decade as high-molar-mass materials by the rhodium- and iron-catalyzed dehydrogenation of primary phosphine-boranes RPH_2_⋅BH_3_.[15] Studies of the coordination chemistry of phosphine-borane ligands at d-block metal centers have allowed the elucidation of the fundamental P–B bond-formation processes leading to dehydrogenative oligomerization and polymerization.[16] These have revealed a twofold role for P–H bonds: activation of the P–H bond by the metal centers to form metal-phosphidoborane intermediates, and promotion of the dehydrogenative coupling of P–H (protic H) with B–H bonds (hydridic H) to release H_2_ and form a P–B bond.[15g, 16] However, as P–H bonds are effectively nonpolar (electronegativity: P=2.19, H=2.20),[9] catalytic dehydrocoupling routes have relied on the electron-withdrawing effect of aryl groups on phosphorus to promote the reaction. This has resulted in relatively limited substrate scope. Thus, the only examples of poly(alkylphosphinoboranes) are of modest molar mass and have been prepared by the slow dehydrocoupling of *i*BuPH_2_⋅BH_3_[15c] and FcCH_2_PH_2_⋅BH_3_[15e] at 110–120 °C over 13–18 h in the presence of Rh catalysts in reactions that generally lead to appreciable chain branching and cross-linking, resulting in a very high polydispersity index (PDI) value (e.g. PDI>5).[15c]

A potential avenue to broaden the substrate scope and circumvent the shortcomings of metal-catalyzed dehydropolymerization routes to polyphosphinoboranes would be the implementation of an addition–polymerization strategy. This would require access to suitable monomeric precursors. Significantly, recent progress by Scheer and co-workers has allowed the facile, gram-scale preparation of H_2_P-BH_2_⋅NMe_3_ (**1 a**), a Lewis base stabilized monomeric phosphinoborane.[17, 18] Elimination of the Lewis base should yield a reactive monomeric phosphinoborane [H_2_P-BH_2_] that might be expected to oligomerize and/or polymerize. In order to explore the potential of this new polymerization strategy in detail we also targeted the aryl-substituted analogue Ph_2_P-BH_2_⋅NMe_3_ (**1 b**) and the alkyl-substituted *t*BuPH-BH_2_⋅NMe_3_ (**1 c**). We therefore developed a salt metathesis route as a novel and convenient method for the generation of substituted phosphanylboranes stabilized only by a Lewis base (Scheme 1). Deprotonation of the corresponding phosphines and subsequent reaction with IBH_2_⋅NMe_3_ afforded the desired phosphanylboranes in good yield and with high purity. Adducts **1 b** and **1 c** were obtained as white solids that are soluble in THF, toluene, Et_2_O, and MeCN and, in the case of **1 c**, also *n*-hexane. Characterization was achieved by multinuclear NMR spectroscopy and single-crystal X-ray diffraction studies (Figure 1).

**Figure 1:**
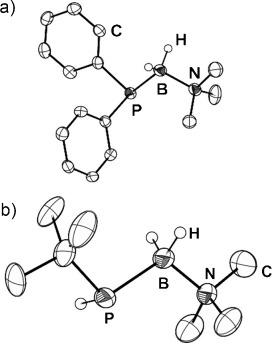
Solid-state structure of 1 b (a) and 1 c (b); ellipsoids at the 50 % probability level. Hydrogen atoms bound to carbon atoms are omitted for clarity. Selected bond lengths [Å] and angles [°]: a): P–B 1.975(2), B–N 1.619(3); P-B-N 112.4(2). b): P–B 1.985(2), N–B 1.621(2); B-P-C 102.7(1), P-B-N 108.9(1).

**Scheme 1:**
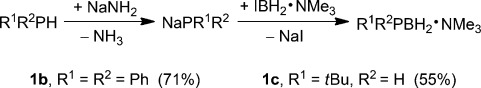
Synthesis of Lewis base stabilized organosubstituted phosphanylboranes 1 b,c.

Attempts to thermally induce oligomerization and polymerization (Scheme 2) were initially made for **1 a** and involved reactions at 80 °C both in the presence and absence of solvent. However, irrespective of the conditions, in the case of this precursor the major fraction of the product (**3 a**) was insoluble in common solvents and the soluble fraction appeared to consist of low-molar-mass, potentially branched oligomers with multiple phosphorus and boron environments.

**Scheme 2:**
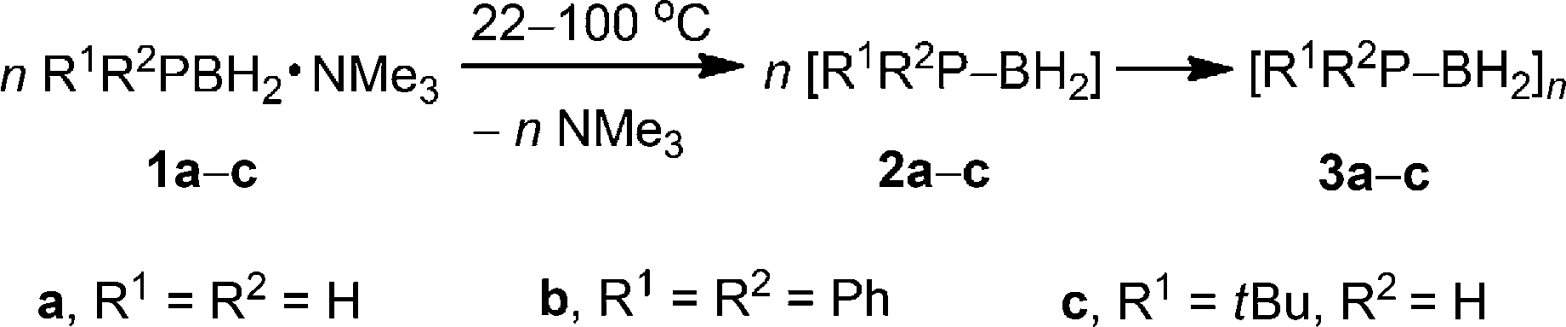
Polymerization/oligomerization of Lewis base stabilized phosphanylboranes (1 a–c).

For example, thermolysis of **1 a** in toluene (80 °C, 20 h) gave a white, waxlike product. The soluble extract in dilute C_6_D_6_[20] gave a ^31^P{^1^H} NMR spectrum that featured a set of three broad signals at *δ*=−110, −116, and −133 ppm, which showed further broadening in the ^1^H-coupled ^31^P NMR spectrum. These resonances are in a similar chemical shift range to those reported for [H_2_P-BH_2_]_x_ prepared via B(C_6_F_5_)_3_-catalyzed dehydrocoupling of H_3_P⋅BH_3_ (*δ*(^31^P)=−95 to −120 ppm), where a mixture of oligomers and low-molar-mass polymer (*M*_n_<2000 g mol^−1^) was formed.[19] Furthermore, one of the peaks has a chemical shift similar to that for the borane complex of **1 a**, BH_3_-H_2_P-BH_2_⋅NMe_3_ (^31^P NMR: *δ*=−116.0),[17a] in which the phosphorus center would exist in a similar environment. The ^11^B{^1^H} spectrum showed a set of three overlapping triplets at roughly *δ*=−38, −40, and −41 ppm as major peaks (^1^*J*_BP_≈65 Hz) which further split into triplets on ^1^H coupling (^1^*J*_BH_≈105 Hz, typical for BH_2_ groups). The ^11^B NMR chemical shifts were similar to those reported for internal BH_2_ groups in phosphinoborane polymers and oligomers ([H_2_P-BH_2_]_*x*_ *δ*(^11^B)=−32 ppm,[19] [PhPH-BH_2_]_*n*_ *δ*(^11^B)=−34.7 ppm).[15a] Several signals at *δ*=−8 to −10 ppm were tentatively assigned to the NMe_3_-coordinated BH_2_ end groups (cf. NMe_3_-capped terminal BH_2_ group in **1 a** at *δ*(^11^B)=−6.7 ppm).[17a] Analysis of the soluble fraction of **3 a** by mass spectrometry (MS) and dynamic light scattering (DLS) was also consistent with the presence of oligomers. For example, electrospray ionization (ESI) MS showed a pattern with intervals of Δ(*m*/*z*)=46, expected for a [H_2_P-BH_2_] moiety, up to 1700 Da, corresponding to up to ca. 37 repeat units (see Figure S12).

As a result of the insolubility of the polyphosphinoborane **3 a** formed from heating **1 a**, we next turned our attention to the analogous thermally induced polymerization of phosphanylboranes with organic substituents at phosphorus (**1 b**,**c**) (Scheme 2). Thermolysis of phosphanylborane **1 b** was conducted in toluene solution at 100 °C for 18 h. The ^1^H, ^31^P, and ^11^B NMR resonances of the isolated product **3 b** were consistent with the formation of oligomeric species [Ph_2_P-BH_2_]_*x*_ and occurred at chemical shifts similar to those reported for [Ph_2_P-BH_2_]_3_ and [Ph_2_P-BH_2_]_4_.[15b] ESI MS analysis of **3 b** indicated the presence of linear NMe_3_-capped oligomers with a maximum detectable mass of up to 1200 g mol^−1^ corresponding to about six repeat units (Figure S15), slightly greater than that in the reports of Rh^I^-catalyzed dehydrocoupling of Ph_2_PH⋅BH_3_.[15b] In addition, the ESI mass spectrum of **3 b** displayed several peaks corresponding to small, NMe_3_-capped oligomeric units, [Me_3_N⋅BH_2_-Ph_2_P-BH_2_⋅NMe_3_]^+^ and [Me_3_N⋅BH_2_-Ph_2_P-BH_2_-Ph_2_P-BH_2_⋅NMe_3_]^+^. These represent a class of highly stable cationic phosphinoborane chains, whose preparation has recently been reported.[18f] Analysis by DLS was also consistent with the formation of oligomeric products that undergo facile aggregation (see the Supporting Information for details).

Finally, we explored the thermolysis of the *t*Bu-substituted phosphanylborane **1 c** using three methods: heating **1 c** at 40 °C for 48 h in the absence of solvent, stirring a toluene solution of **1 c** at room temperature (22 °C), and performing the latter experiment at 40 °C for 48 h. After complete consumption of the starting material (and removal of the solvent for reactions conducted in toluene), the crude product was dissolved in *n*-hexane and precipitated by adding the resulting solution slowly to vigorously stirred acetonitrile. All three methods led to the isolation of the product **3 c** as a fine white powder (Figure 3, inset) with similar NMR spectra. The ^11^B{^1^H} NMR spectrum featured a single very broad signal at *δ*=−38 ppm. The ^31^P{^1^H} NMR spectrum featured a set of three broad signals at *δ*=−19, −21, and −24 ppm. Further broadening and splitting into poorly defined doublets was observed in the ^1^H-coupled ^31^P NMR spectrum. We attribute the overlapping resonances to tacticity; the tentative assignment of rm, mr, rr, and mm triads is based on statistical probability (Figure 2). Similar features have been observed in poly(methylenephosphine) polymers.[4a] Overall, the observed NMR spectra for **3 c** were similar to those for [RHP-BH_2_]_*n*_ (R=Ph, *i*Bu, *p*-*n*BuC_6_H_4_, *p*-dodecylC_6_H_4_).[15a–c,g]

**Figure 2:**
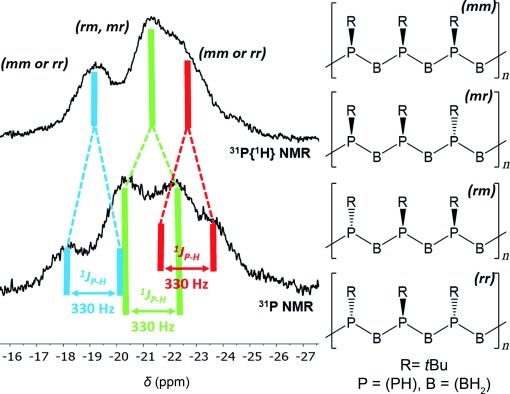
^31^P and ^31^P{^1^H} NMR spectra of [*t*BuPH-BH_2_]_*n*_ (3 c) in CDCl_3_ with proposed tacticity resulting in overlapped resonances.

**Figure 3:**
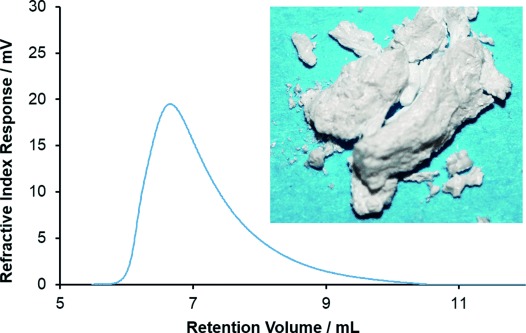
GPC trace for [*t*BuPH-BH_2_]_*n*_ (3 c, from polymerization in toluene, 22 °C, 48 h) in CHCl_3_. Inset: photograph of a purified sample of 3 c.

The ESI mass spectra of acetonitrile solutions of **3 c** (reaction in toluene, 22 °C, 48 h) showed patterns corresponding to the successive loss of Δ(*m*/*z*)=102, characteristic of a single unit of [*t*BuPH-BH_2_] (Figure S19). Samples obtained from the three methods were analyzed by DLS at optimized concentrations in CH_2_Cl_2_. The range of values obtained for *R*_h_ of 4.4–5.5 nm correspond to molar masses of 26 800–39 900 g mol^−1^ for monodisperse polystyrene samples in THF (Figure S20).[21] GPC analysis of the samples with CHCl_3_ as eluent, also using polystyrene standards, was consistent with these results within experimental error and showed the presence of polymer with molar masses (*M*_n_) of 27 800–35 000 g mol^−1^ with polydispersity indices of 1.6–1.9 (Figures 3 and S22).

We propose that polymerization of **1 a**–**c** is triggered by the initial thermolysis of Lewis base stabilized phosphanylboranes **1 a**–**c**, leading to elimination of NMe_3_ to form the unprotected monomeric phosphinoborane intermediates **2 a**–**c**. The resulting absence of the Lewis base leads to a lack of electronic stabilization for **2 a**–**c**. As a result, the lone pair at phosphorus together with a vacant p orbital at boron, in conjunction with the aforementioned electronic destabilization, appears to promote a head-to-tail addition oligomerization/polymerization sequence which ultimately affords **3 a**–**c**, although the full mechanistic details are not yet clear (Scheme 2). We attribute the difference in product distribution to the reactivity of **2 a**–**c** and the solubility of the polymer products **3 a**–**c**. Sterically unencumbered **2 a** is likely to be highly reactive and forms the insoluble material, which may be of high molar mass, together with soluble oligomers. In contrast, **2 b**, which contains two bulky phenyl groups at phosphorus, appears to afford only oligomers. Presumably the steric bulk hinders polymer formation both kinetically, and possibly thermodynamically as well. In contrast, the *tert*-butyl-substituted species **3 c** affords soluble, high-molecular-weight polymer.

High-molar-mass poly(phenylphosphinoborane) free of cross-linked material has been recently prepared using an iron-based dehydrocoupling catalyst in toluene solution, a reaction significantly more efficient that the previously reported Rh-catalyzed process performed in the absence of solvent.[15g] We were intrigued whether alkyl-substituted polymer **3 c** would be accessible by a similar route. For comparison we prepared poly(phenylphosphinoborane) from PhPH_2_⋅BH_3_ and 1 mol % [Cp(CO)_2_Fe(OSO_2_CF_3_)] (100 °C, 24 h) and isolated the material with *M*_n_=59 000 g mol^−1^ and PDI=1.6.[15g] When *t*BuPH_2_⋅BH_3_ was treated with [Cp(CO)_2_Fe(OSO_2_CF_3_)] under the same conditions (Scheme 3) near complete consumption of *t*BuPH_2_⋅BH_3_ required 176 h by ^31^P and ^11^B NMR analysis. Subsequent precipitation into, and washes with cold pentane afforded a dark amber waxlike product. The ^31^P{^1^H}/^31^P and ^11^B{^1^H} NMR spectra featured multiple broad overlapping resonances (*δ*(^11^B)=−40 ppm, *δ*(^31^P)≈−20 ppm). Although ESI MS showed peaks separated by Δ(*m/z*) = 102, attributed to units of [*t*BuPH-BH_2_], masses up to only 1100 Da were detected. Moreover, GPC analysis of the products with CHCl_3_ as eluent revealed no high-molar-mass component and the product appears to be an oligomer of 10 units or less. This is in stark contrast to the high-molar-mass polymer (**3 c**) obtained via the thermally induced polymerization of phosphanylborane **1 c**.

**Scheme 3:**
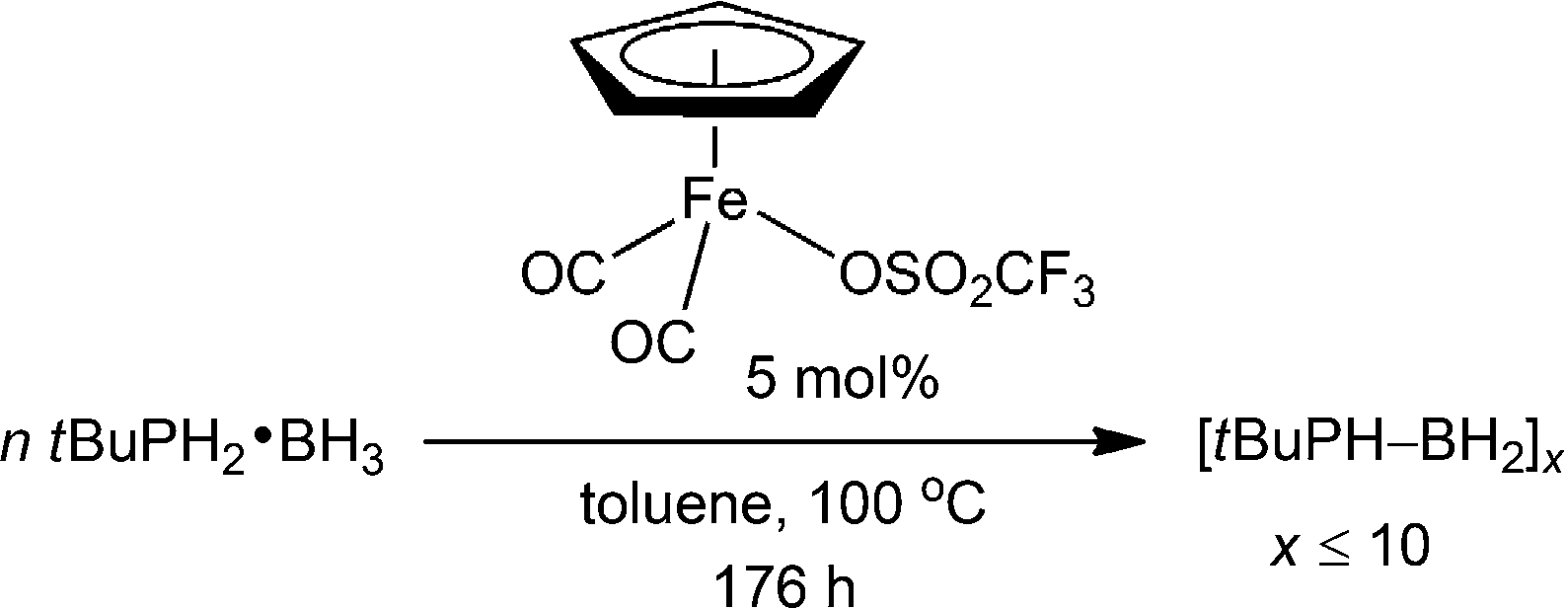
Attempted synthesis of [*t*BuPH-BH_2_]_*n*_ (3 c) via catalytic dehydrocoupling of *t*BuPH_2_⋅BH_3_.

In summary, a straightforward synthesis of organosubstituted monomeric phosphanylboranes stabilized only by a Lewis base has been developed to obtain compounds **1 b** and **1 c**. Simple thermal treatment of the monomeric Lewis base stabilized phosphinoboranes **1 a–c** led to the formation of oligomeric and polymeric compounds **3 a–c**. Due to the low solubility of **3 a**, characterization of this polymer was limited. Polymerization of **1 b** led to short-chain oligomers **3 b** which could be characterized by multinuclear NMR spectroscopy and mass spectrometry. However, polymerization of **1 c** afforded **3 c** with high molar mass (*M*_n_=27 800–35 000 g mol^−1^) and reasonably low PDI (1.6–1.9) characteristic of a mainly linear material. In contrast, previous work with Rh catalysts has given lower-molar-mass, branched materials (*M*_n_<ca. 10 000 g mol^−1^) under forcing thermal conditions in the melt where the yields have been limited by gel formation.[15c]In addition, polyphosphinoborane **3 c** could not be accessed via the recently reported Fe-catalyzed catalytic dehydrocoupling route, presumably also due to the deactivated P–H bond in the alkylphosphinoborane monomer.

Based on these results, the new metal-free polymerization method described offers considerable promise for the preparation of a range of new polyphosphinoboranes with alkyl substituents on phosphorus that are of interest as elastomers, flame-retardant materials, and ceramic precursors. Expansion of the substrate/polymer scope, optimization of the reaction conditions, and the detailed elucidation of the reaction mechanism, which appears to involve the addition/head-to-tail polymerization of transient phosphinoborane monomers, are topics currently under investigation.

## Supporting Information

As a service to our authors and readers, this journal provides supporting information supplied by the authors. Such materials are peer reviewed and may be re-organized for online delivery, but are not copy-edited or typeset. Technical support issues arising from supporting information (other than missing files) should be addressed to the authors.

miscellaneous_information
