# Supplementary material for: Metal-Free Addition/Head-to-Tail Polymerization of Transient Phosphinoboranes, RPH-BH2: A Route to Poly(alkylphosphinoboranes)
Source: Angew Chem Int Ed Engl. 2015 Oct 2;54(46):13782–6. doi: 10.1002/anie.201507084 (PMC4648028; doi:10.1002/anie.201507084)
Supplement: Supplementary file 1 — miscellaneous_information [file anie0054-13782-sd1.pdf]

Angewandte Chemie
Eine Zeitschrift der Gesellschaft Deutscher Chemiker

Supporting Information

Metal-Free Addition/Head-to-Tail Polymerization of Transient Phosphinoboranes, RPH-BH_2: A Route to Poly(alkylphosphinoboranes)

Christian Marquardt, Titel Jurca, Karl-Christian Schwan, Andreas Stauber, Alexander V. Virovets, George R. Whittell, Ian Manners, and Manfred Scheer**

anie_201507084_sm_miscellaneous_information.pdf

CONTENTS

General Experimental Section	S2-S3
Synthesis and Characterization of **1b**, **1c,** and $^{t}BuPH_2{\cdot}BH_3$	S3-S6
Thermolysis-induced polymerization of **1a-c** to form **3a-c**	S6-S15
Attempted Fe-Catalyzed dehydrocoupling of $^{t}BuPH_2{\cdot}BH_3$	S16-S18
Crystallographic details for **1b** and **1c**	S19-S20
References	S21

Experimental Section

General Experimental:

Unless otherwise noted, all manipulations were performed under an atmosphere of dry argon using standard glove-box and Schlenk techniques. All solvents were degassed and purified by standard procedures. The compounds $IBH_2{\cdot}NMe_3$,[1] $H_2PBH_2{\cdot}NMe_3$ (**1a**),[2] and $^tBuPH_2$[3] were prepared according to literature procedures. Other chemicals were obtained from Sigma-Aldrich ($NaNH_2$) or STREM Chemicals, INC. (PPh_2H).

The NMR spectra for the monomers **1b, 1c** and for the compounds **3b** and **3c** were recorded on an Avance 400 spectrometer (^{1}H: 400.13 MHz, ^{31}P: 161.976 MHz, ^{11}B: 128.378 MHz, ^{13}C{^{1}H}: 100.623 MHz) with δ [ppm] referenced to external $SiMe_4$ (^{1}H, ^{13}C), H_3PO_4 (^{31}P), $BF_3{\cdot}Et_2O$ (^{11}B). Other NMR spectra were recorded using Oxford Jeol Eclipse 300 and 400 MHz spectrometers. ^{1}H NMR spectra were calibrated using residual proton signals of the solvent: (δ ^{1}H($CHCl_3$) = 7.24; δ ^{1}H(C_6D_5H) = 7.20). ^{13}C NMR spectra were calibrated using the solvent signals (δ ^{13}C($CDCl_3$) = 77.0; δ ^{13}C(C_6D_6) = 128.0). ^{11}B and ^{31}P NMR spectra were calibrated against external standards (^{31}P: 85% H_3PO_4(aq) (δ ^{31}P = 0.0); ^{11}B: $BF_3{\cdot}OEt_2$ (δ ^{11}B = 0.0)).

IR spectra were measured on a DIGILAB (FTS 800) FT-IR spectrometer. Mass spectra were recorded on a ThermoQuest Finnigan TSQ 7000 (ESI-MS) or a Finnigan MAT 95 (FD-MS and EI-MS) or a Bruker Daltonics Apex IV Fourier transform Ion Cyclotron mass spectrometer. The C, H, N analyses were measured on an Elementar Vario EL III apparatus. Gel permeation chromatography (GPC) was performed on a Viscotek RImax chromatograph, equipped with an automatic sampler, a pump, an injector and inline degasser. The columns were contained within an oven (35°C) and consisted of styrene/divinyl benzene gels with pore sizes ranging from 500 Å to 100,000 Å. THF containing 0.1 % w/w [$n$$Bu_4N$]Br and 1% v/v toluene was used as the eluent at a flow rate of 1.0 mL min^{-1}. All samples analysed by GPC were dissolved in the eluent (2 mg mL^{-1}), stirred for 1 h at room temperature and passed through a membrane filter (200 nm pores) before analysis. The calibration was conducted using a series of monodisperse polystyrene standards obtained from Aldrich. Dynamic Light Scattering (DLS) was performed on a Malvern Instruments Zetasizer Nano S using a 5mW He-Ne laser (633 nm). The correlation function was collected in real time and fitted with a function capable of modelling polymodal size distributions.

The single crystal X-Ray structure analysis was performed on a Gemini R Ultra CCD diffractometer from Agilent Technologies (formerly Oxford Diffraction) applying Cu-K_α radiation (λ = 1.54178 Å) at 123 K. Crystallographic data are given in Tables S1. Absorption corrections were applied semi-empirically from equivalent reflections or analytically (SCALE3/ABSPACK algorithm implemented in CrysAlis PRO software by Agilent Technologies Ltd).[4] All structures were solved using SIR97[5], and refined using SHELXL-14.[6] The hydrogen positions of the methyl groups were located geometrically and refined riding on the carbon atoms. Hydrogen atoms belonging to BH_2 and PH_2 groups were located from the difference Fourier map and refined without constraints. Due to the bad displacement parameters of the heavily disordered ^{t}Bu-moiety in **1c**, the carbon atoms were

refined applying ISOR restraints. Figures were created with OLEX.[7] All cif-files are available online from the Cambridge Crystallographic Data Centre.

Synthesis of Ph_2P-BH_2·NMe_3 (1b):

1.740 mL (1.866 g, 10 mmol) Ph_2PH was added to a suspension of 390 mg (10 mmol) $NaNH_2$ in 20 ml THF at -40 °C. The mixture was allowed to reach room temperature and was stirred for an additional 2 h, until a clear red solution was obtained. After cooling to -80 °C, the $NaPPh_2$ solution was added to a solution of 1.990 g (10 mmol) IBH_2·NMe_3 in 20 mL THF at -80 °C. After warming up to room temperature, the mixture was stirred for an additional 18 h. All volatiles were removed under vacuum and the remaining solids were suspended in 50 mL of toluene and filtered over diatomaceous earth. The toluene was removed, and the resulting white solid washed 5 times with 10 mL of Et_2O at 0 °C to remove NaI. The remaining white solid was dissolved in 20 mL of toluene and filtered over diatomaceous earth. After removing the toluene the resulting **1b** was washed 3 times with 10 mL of *n*-hexane and dried under vacuum. **1b** is a white powder at room temperature. Crystals of **1b** were obtained by dissolving a small amount of **1b** in *n*-hexane and storing the solution at 3°C. Yield of (**1b**): 1.833 g (71 %); ^{1}H NMR (400 MHz, C_6D_6): δ = 1.92 (s, 9H, NMe_3), 2.84 (q, $^1J_{H,B}$ = 107 Hz, 2H, BH_2), 7.07 (m, 2H, *p*-Ph), 7.20 (m, 4H, *m*-Ph) 7.87 (m, 4H, *o*-Ph). ^{31}P NMR (162 MHz, C_6D_6): δ = –39.5 (m, br, PPh_2). ^{31}P{^{1}H} NMR (162 MHz, C_6D_6): δ = –39.5 (m, br, PPh_2). ^{11}B NMR (128 MHz, C_6D_6): δ = –1.7 (td, $^1J_{B,P}$ = 45 Hz, $^1J_{B,H}$ = 107 Hz, BH_2). ^{11}B{^{1}H} NMR (128 MHz, C_6D_6): δ = –1.7 (d, $^1J_{B,P}$ = 45 Hz, BH_2). ^{13}C NMR (101 MHz, C_6D_6): δ = 52.4 (d, $^3J_{P,C}$ = 11 Hz, NMe_3), 126.44 (s, *p*-Ph), 128.13 (d, $^3J_{P,C}$ = 6 Hz, *m*-Ph) 134.80 (d, $^2J_{P,C}$ = 16 Hz, *o*-Ph), 143.78 (d, $^1J_{P,C}$ = 16 Hz, *i*-Ph). IR (KBr): $\tilde{\nu}$ = 3061 (w), 3040 (w), 3010 (w), 2994 (w), 2943 (w), 2918 (w), 2361 (s, br, BH), 2290 (w, br, BH), 1580 (w), 1478 (s), 1465 (s), 1442 (s), 1430 (m), 1403 (w), 1315 (w), 1252 (m), 1155 (m), 1123 (s), 1078 (m), 1058 (s), 1027 (m), 1015 (w), 850 (s), 747 (s), 699 (s), 639 (w), 510 (m), 473 (w). EI-MS (toluene): *m/z* = 72 (42 %, $[Me_3N\cdot BH_2]^+$), 108 (100 %, $[PhP]^+$), 257 (22.5 %, $[Me_3N\cdot BH_2–PPh_2]^+$). Elemental analysis (%) calculated for $C_{15}H_{21}BNP$ (**1b**): C: 70.07, H: 8.23, N: 5.45; found: C: 70.05, H: 8.11, N: 5.41.

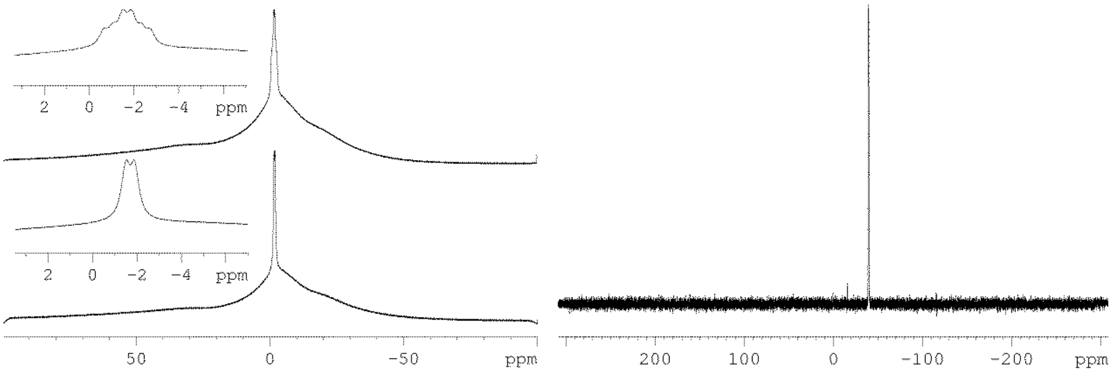

Figure S1: (*bottom left*) ^{11}B{^{1}H} (128 MHz), (*top left*) ^{11}B (128 MHz), (*right*) ^{31}P (162 MHz) NMR spectra of **1b** in in C_6D_6.

Synthesis of ^{t}BuHP-BH$_2$·NMe$_3$ (1c):

2.550 mL (1.802 g, 20 mmol) ^{t}BuPH$_2$ was added to a suspension of 780 mg (20 mmol) $NaNH_2$ in 20 ml THF at -40 °C. The mixture was allowed to reach room temperature and was stirred for an additional 2 h, until a clear yellow solution was obtained. After cooling to -80 °C, the Na^{t}BuPH solution was added to a solution of 3.980 g (20 mmol) IBH$_2$·NMe$_3$ in 20 mL THF at -80 °C. After warming up to room temperature, the mixture was stirred for an additional 18 h. All volatiles were removed under vacuum and the remaining solids suspended in 20 mL of *n*-hexane and filtered over diatomaceous earth. The *n*-hexane was removed, and then **1c** was purified by sublimation at 45 °C ($1·10^{-3}$ mbar). **1c** is a white powder at room temperature. Crystals of **1c** were obtained by dissolving a small amount of **1c** in *n*-hexane and storing the solution at -28 °C. Yield of (**1c**): 1.767 g (55 %); ^{1}H NMR (400 MHz, C_6D_6): δ = 1.50 (dm, $^{3}J_{H,P}$ = 11 Hz, 9H, P^{t}Bu), 1.94 (s, 9H, NMe$_3$), 2.60 (d, $^{1}J_{H,P}$ = 197 Hz, 1H, PH^{t}Bu), 2.67 (q, BH$_2$). ^{31}P NMR (162 MHz, C_6D_6): δ = –67.6 (d, br $^{1}J_{H,P}$ = 197 Hz, PH). ^{31}P{^{1}H} NMR (162 MHz, C_6D_6): δ = –67.6 (q, $^{1}J_{B,P}$ = 48 Hz, PH). ^{11}B NMR (128 MHz, C_6D_6): δ = –6.0 (td, $^{1}J_{B,P}$ = 48 Hz, $^{1}J_{B,H}$ = 104 Hz, BH$_2$). ^{11}B{^{1}H} NMR (128 MHz, C_6D_6): δ = –6.0 (d, $^{1}J_{B,P}$ = 48 Hz, BH$_2$). ^{13}C NMR (101 MHz, C_6D_6): δ = 26.4 (s, C), 33.3 (d, $^{2}J_{C,P}$ = 11 Hz, ^{t}Bu), 52.0 (d, $^{3}J_{C,P}$ = 11 Hz, NMe$_3$). IR (KBr): $\tilde{\nu}$ = 2993 (m), 2962 (m), 2939 (s), 2892 (m), 2854 (m), 2388 (s, br, BH), 2295 (w, br, BH), 2244 (m, PH), 1482 (s), 1464 (s), 1403 (w), 1358 (m), 1252 (m), 1189 (w), 1154 (s), 1124 (s), 1067 (s), 1012 (w), 984 (w), 872 (m), 847 (s), 846 (s), 693 (w), 545 (w), 599 (w), 475 (w). EI-MS (solid): *m/z* = 58 (11 %, [Me$_2$NCH$_2$]$^{+}$), 72 (100 %, [Me$_3$N·BH$_2$]$^{+}$), 104 (1.8 %, [Me$_3$N·BH$_2$–PH]$^{+}$), 161 (6.4 %, [Me$_3$N·BH$_2$–PH^{t}Bu]$^{+}$). Elemental analysis (%) calculated for $C_7H_{21}BNP$ (**1c**): C: 52.13, H: 13.13, N: 8.69; found: C: 52.32, H: 13.06, N: 8.70.

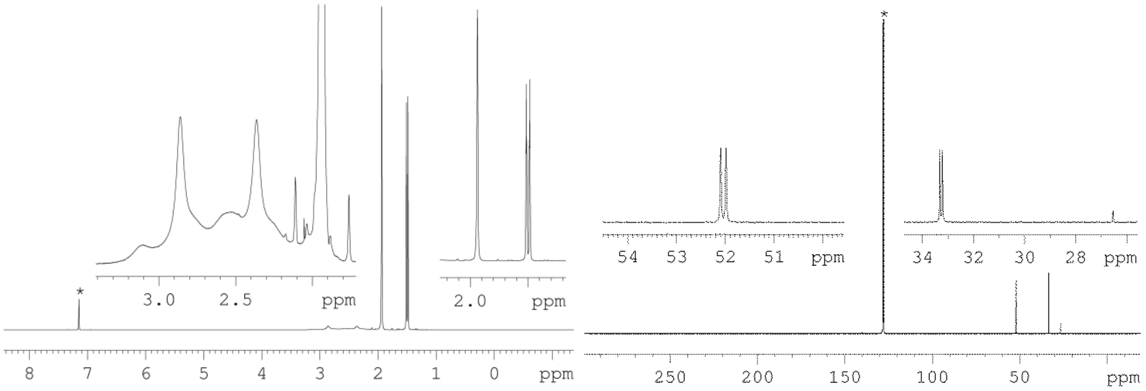

Figure S2: (*left*) ^{1}H (400 MHz); (*right)* ^{13}C{^{1}H} (101 MHz) NMR spectra of **1c** in in C_6D_6.* = solvent (C_6D_6)

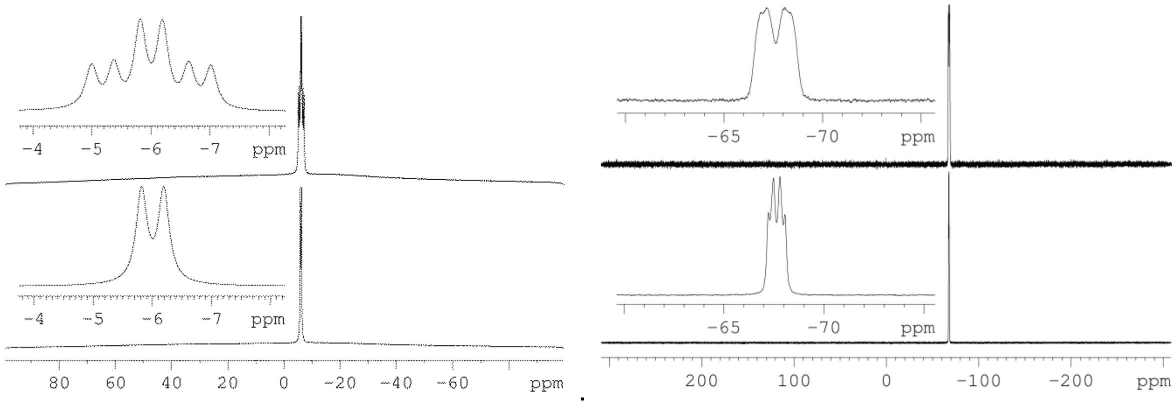

Figure S3: (*bottom left*) $^{11}B\{^1H\}$ (128 MHz), (*top left*) ^{11}B (128 MHz), (*bottom right*) $^{31}P\{^1H\}$ (162 MHz), (*top right*) ^{31}P (162 MHz) NMR spectra of **1c** in in C_6D_6.

Synthesis of ^{t}BuPH$_2$·BH$_3$:

Under flow of N_2, a solution of ^{t}Bu$_2$PCl$_2$ (1.09 g, 6.8 mmol) in dibutyl ether (5 mL) was added dropwise to a dibutyl ether (20 mL) suspension of $LiBH_4$ (0.30 g, 14 mmol) cooled to 5 ^oC with an ice bath. The mixture became cloudy immediately and was allowed to stir for 30 min. The reaction flask was then assembled into a short-path distillation setup and reaction solution held at -5 ^oC while the receiving flask was held at -196 ^oC in liquid N_2. Upon placing the system under static vacuum, ^{t}BuPH$_2$·BH$_3$ was transferred to the receiving flask as a clear oil. The resulting lower yield of 437 mg (62 %) is a result of loss of volatile product under vacuum. ^1H NMR (300 MHz, toluene-d8): δ = 3.75 (br doublet of quartets, P*H*, $^1J_{P,H}$ = 350 Hz), 0.85 (d, ^{t}Bu*H*, $^3J_{P,H}$ = 15 Hz), 0.4-1.8 (br m, B*H*); ^{31}P NMR (121 MHz, toluene-d8): δ -11.5 (br triplet of multiplets, *P*H$_2$, $^1J_{P,H}$ = 350 Hz; $^{31}P\{^1H\}$, (q, $^1J_{P,B}$ = 34 Hz); $^{11}B\{^1H\}$ NMR (96 MHz, toluene-d8): δ -44.3 (br d, *B*H$_3$, $^1J_{B,P}$ = 33 Hz). The resulting product was used for catalytic dehydrocoupling experiments without further purification.

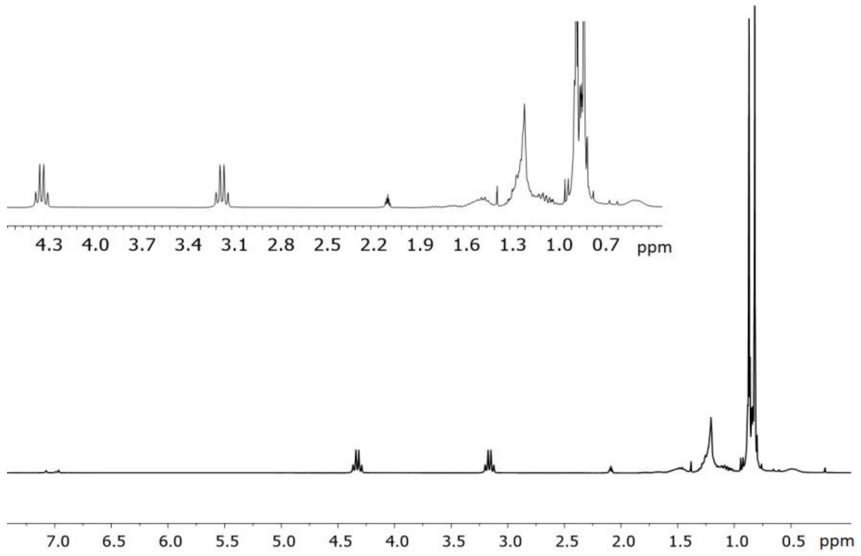

Figure S4: ^1H (300 MHz) NMR spectra of ^{t}BuPH$_2$·BH$_3$ in toluene-d8.

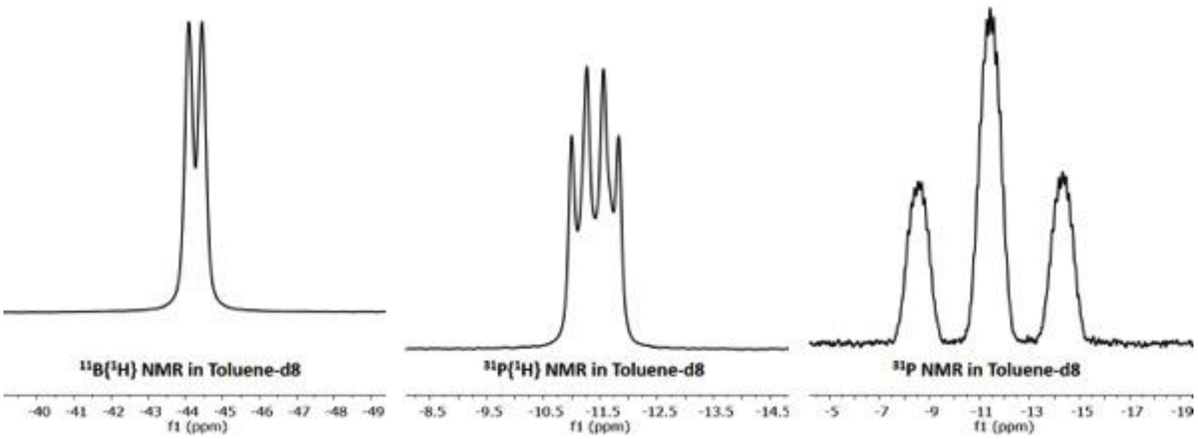

Figure S5: (*left*) $^{11}B\{^1H\}$ (96 MHz), *(middle)* $^{31}P\{^1H\}$ (121 MHz), *(right)* ^{31}P (121 MHz) NMR spectra of $^tBuPH_2{\cdot}BH_3$ in toluene-d8.

Polymerization Experiments:

Polymerization of H_2P-$BH_2{\cdot}NMe_3$ (1a) to form $[H_2P–BH_2]_n$ (3a)

a) 0.300 mL (262 mg, 2.5 mmol) of H_2P-$BH_2{\cdot}NMe_3$ was dissolved in 10 mL of toluene and heated at 80 °C for 20 h. All volatiles were removed under vacuum to give a white residue with an oily to wax like consistency which was nearly insoluble in all common solvents. Efforts were carried out to purify $[PH_2–BH_2]_n$ by extraction with different solvents but were not successful. Extracts in different solvents were analysed by ESI-MS, EI-MS, NMR and DLS. Yield of (**3a**): no reliable determination was possible, as the polymer contains solvent and trace starting material. $^{31}P\{^1H\}$ NMR (121 MHz, C_6D_6): δ = –109.7 (br), –115 (br), –132.5 (br). ^{11}B NMR (96 MHz, C_6D_6): δ = –38 to –41 (m, br, BH_2), –8 to –10 (td, br, BH_2). $^{11}B\{^1H\}$ NMR (96 MHz, C_6D_6): δ = –38 to –41 (m, br, BH_2), –8 to –10 (d, br, BH_2).

b) 0.300 mL (262 mg, 2.5 mmol) of H_2P-$BH_2{\cdot}NMe_3$ was heated at 80 °C for 70 h, affording a white waxy residue which was nearly insoluble in all common solvents. Efforts to purify $[PH_2–BH_2]_n$ by extraction with different solvents were not successful. Analysis of **3a** was conducted by ESI-MS, EI-MS, NMR and DLS. Yield of (**3a**): 86 mg (75 %) ^1H NMR (300 MHz, C_6D_6): δ = 1.8 (br, BH_2), 3.6 (br, PH_2). $^{31}P\{^1H\}$ NMR (121 MHz, C_6D_6): δ = –112 to –105 (br), –125 to –120 (br), –132 (br), –142 (br). ^{11}B NMR (96 MHz, C_6D_6): δ = –34 to –42 (m, br, BH_2), –6 to –12 (td, br, BH_2). $^{11}B\{^1H\}$ NMR (96 MHz, C_6D_6): δ = –34 to –42 (m, br, BH_2), –6 to –12 (d, br, BH_2).

ESI-MS of **3a** (obtained from polymerization reaction in toluene, 80 ^oC, 20 h) in acetonitrile showed signals up to 1700 Da. A systematic loss of 46 m/z, consistent with $[H_2P$-$BH_2]$ was observed (Figure S12). The polymer consists of at least 37 repeat units, however no exact end groups could be determined by ESI-MS.

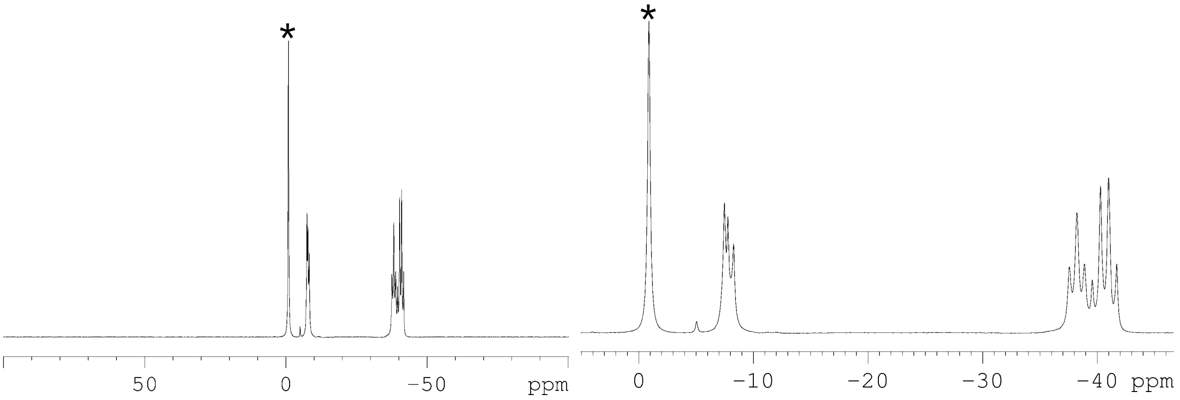

Figure S6: $^{11}B\{^{1}H\}$ (96 MHz) NMR spectra of **3a** (from polymerization reaction in toluene, 80 ^{o}C, 20 h) in C_6D_6; * = $ClBH_2{\cdot}NMe_3$ (starting material for **1a**).

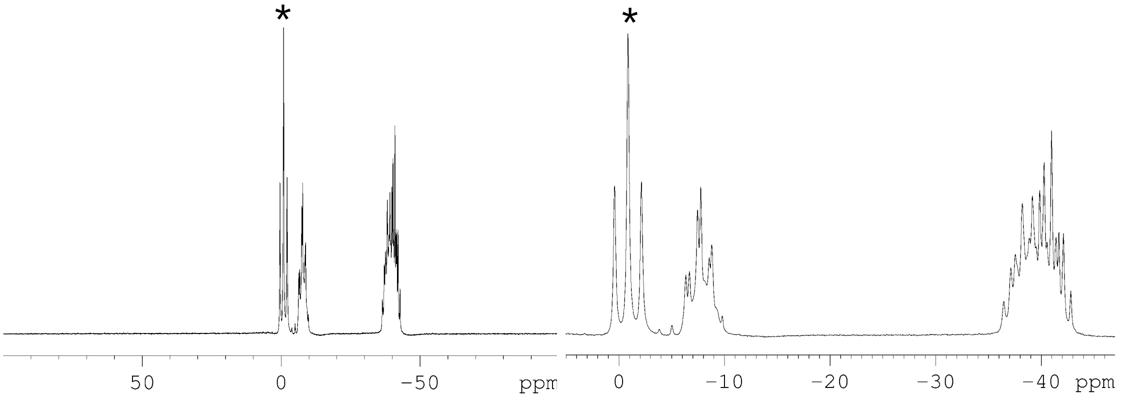

Figure S7: ^{11}B (96 MHz) NMR spectra of **3a** (from polymerization reaction in toluene, 80 ^{o}C, 20 h) in C_6D_6; * = $ClBH_2{\cdot}NMe_3$ (starting material for **1a**).

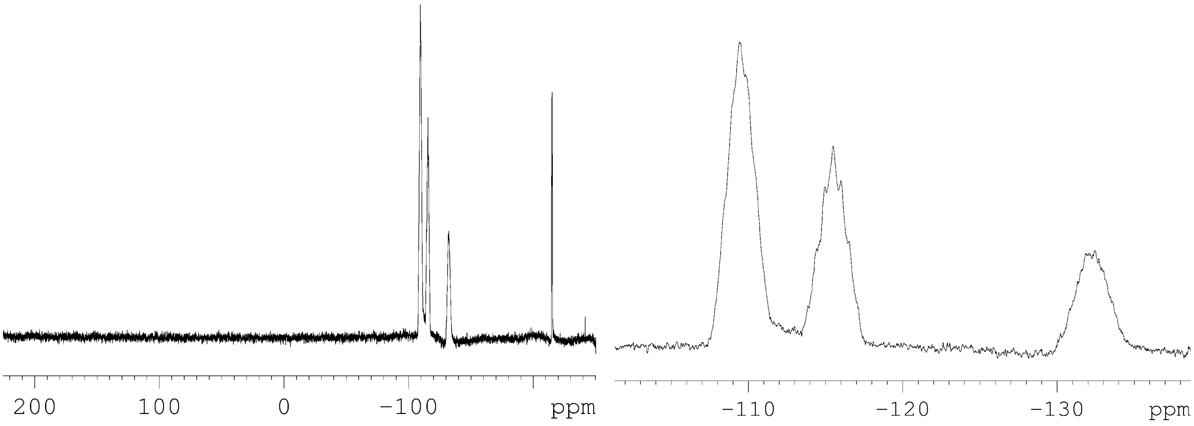

Figure S8: $^{31}P\{^{1}H\}$ (121 MHz) NMR spectra of **3a** (from polymerization reaction in toluene, 80 ^{o}C, 20 h) in C_6D_6.

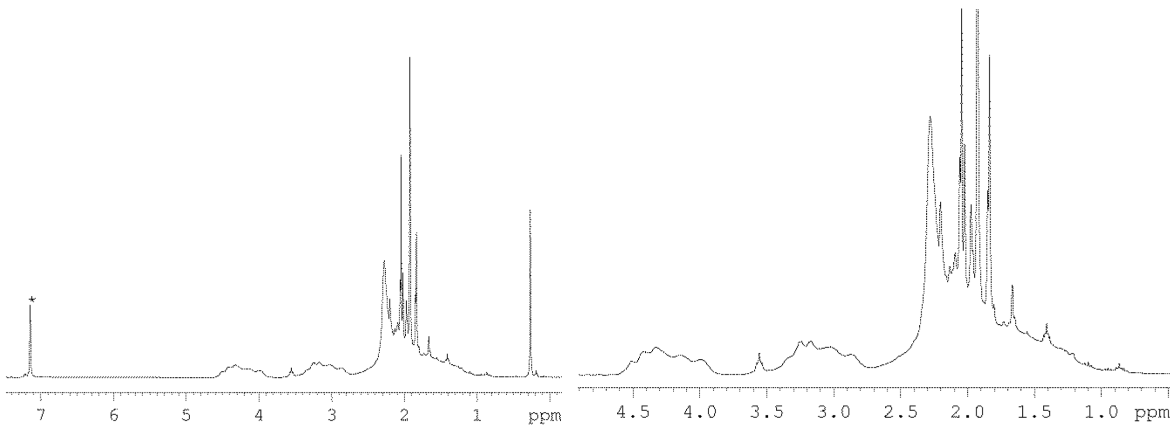

Figure S9: ^{1}H (300 MHz) NMR spectra of **3a** (from neat polymerization reaction, 80 °C, 70 h) in C_6D_6; * = solvent (C_6D_6).

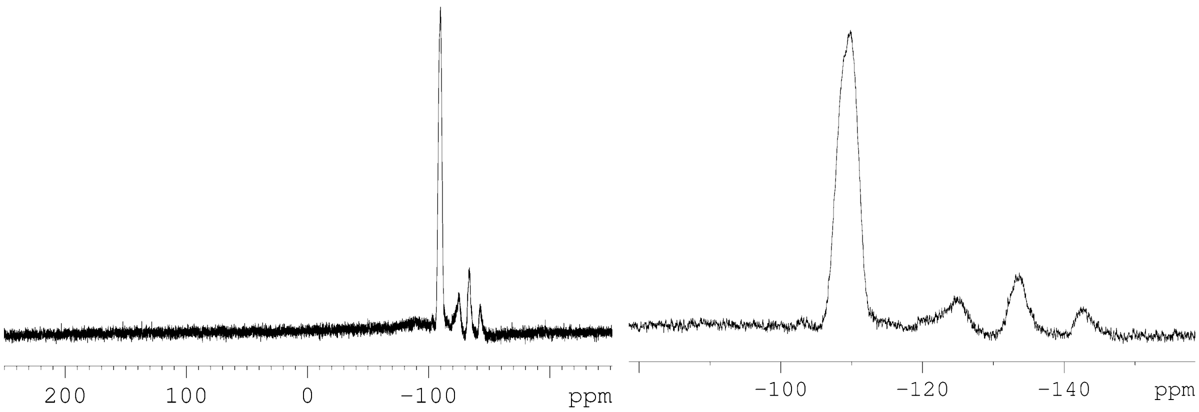

Figure S10: $^{31}P\{^{1}H\}$ (121 MHz) NMR spectra of **3a** (from neat polymerization reaction, 80 °C, 70 h) in C_6D_6.

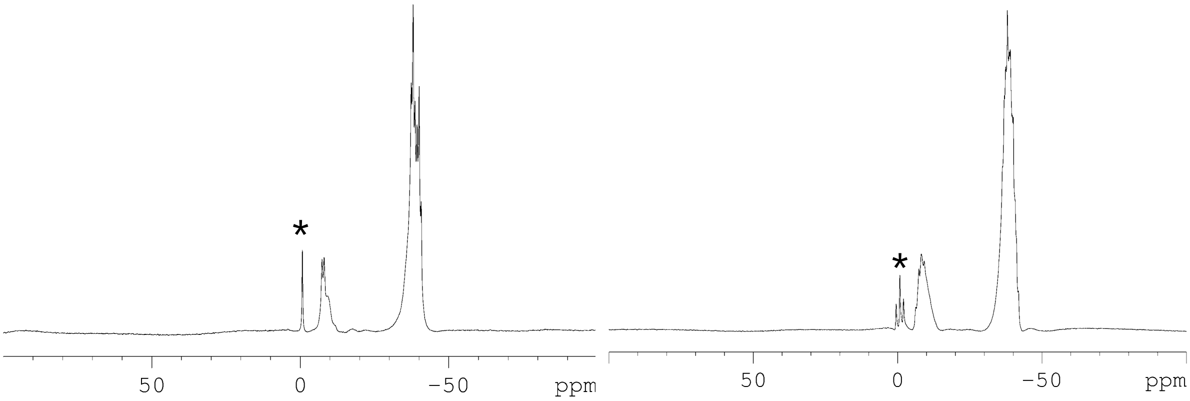

Figure S11: (*left*) $^{11}B\{^{1}H\}$ (96 MHz), (*right*) ^{11}B (96 MHz) NMR spectra of **3a** (from neat polymerization reaction, 80 °C, 70 h) in C_6D_6; * = $ClBH_2{\cdot}NMe_3$ (starting material for **1a**).

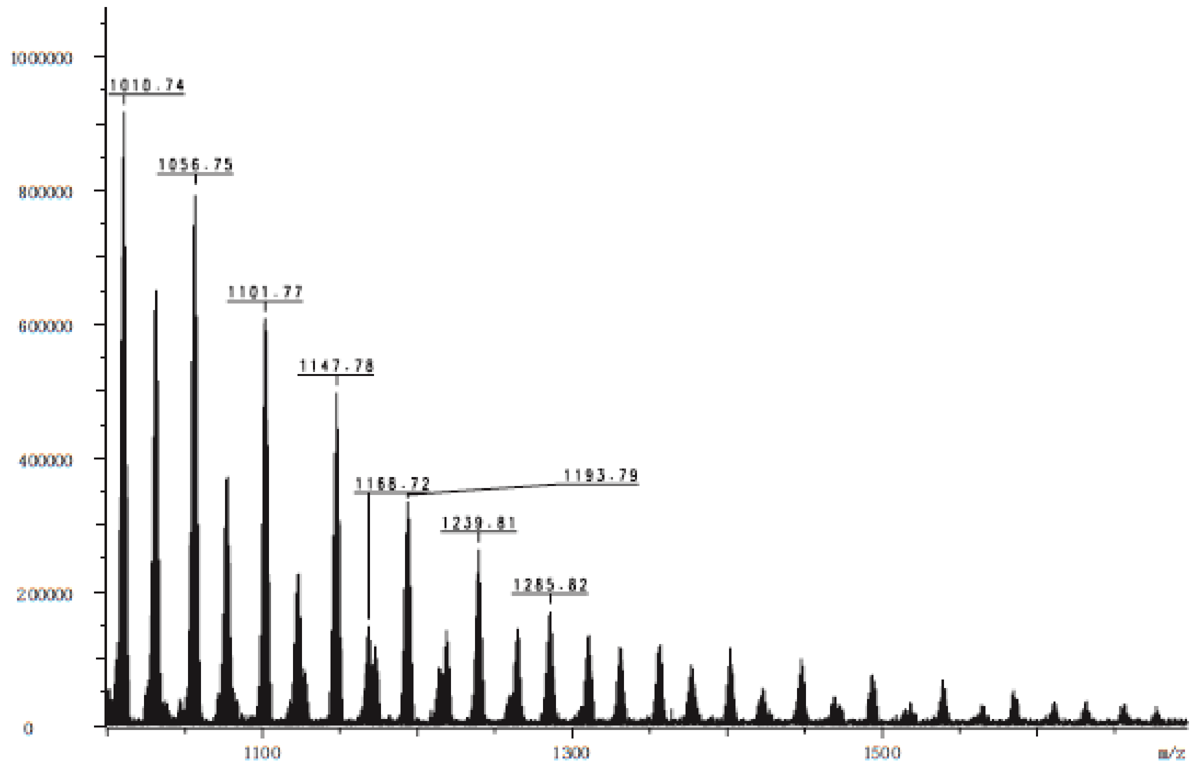

Figure S12: ESI – mass spectrum of **3a** (from polymerization reaction in toluene, 80 ^{o}C, 20 h) in acetonitrile.

Attempted Polymerization of Ph_2P-BH_2·NMe_3 (1b) to form [Ph_2P–BH_2]$_n$ (3b)

600 mg (2.33 mmol) of Ph_2P-BH_2·NMe_3 was dissolved in 20 mL toluene and heated to 100 °C for 18 h. All volatiles were removed under vacuum affording a fine white powder (**3b**). NMR measurement of a sample showed that the reaction proceeded cleanly, so no further purification was conducted prior to analysis. Yield of (**3b**): 402 mg (86 %) ^{31}P NMR (162 MHz, THF-d8): δ = –18.5 (s, br, P). ^{31}P{^{1}H} NMR (162 MHz, THF-d8): δ = –18.5 (s, br, P). ^{11}B NMR (128 MHz, THF-d8): δ = –33.7 (br, BH_2). ^{11}B{^{1}H} NMR (128 MHz, THF-d8): δ = –33.7 (br, BH_2).

Thermolysis of **1b** in toluene at 100 °C for 18 h led to complete consumption of the starting material and formation of **3b**. The ^{31}P{^{1}H} NMR featured a very broad signal at δ = -18 ppm, which showed further broadening in the ^{31}P NMR spectrum. Generation of a small amount of free phosphine Ph_2PH was also observed. The ^{11}B{^{1}H} NMR spectrum featured a very broad uniform signal at δ = -34 ppm, suggestive of either a low molecular weight cyclic oligomeric species or a high molecular weight polymer whose end-groups cannot be detected. The observed NMR signals were similar to those reported for [Ph_2P–BH_2]$_3$ and [Ph_2P–BH_2]$_4$.[7] ESI-MS of **3b** in both THF and toluene revealed a fragmentation pattern which featured loss of units with 198 m/z (representative of [Ph_2P–BH_2]), and a maximum detectable mass in the region of 1200 Da (Figure S15). This was indicative of NMe_3-capped oligomers with no greater than 6 repeat units.

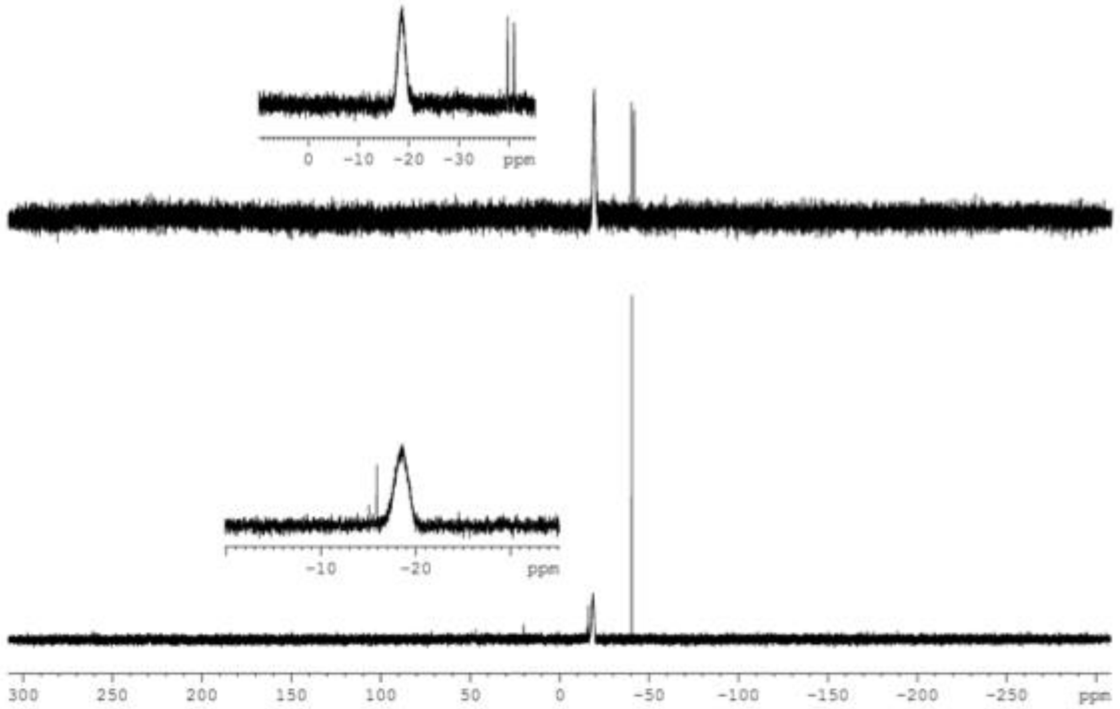

Figure S13: (*bottom*) $^{31}P\{^{1}H\}$ (162 MHz), (*top*) ^{31}P (162 MHz) NMR spectra of **3b** (from polymerization reaction in toluene, 100 ^{o}C, 18 h) in THF-d8.

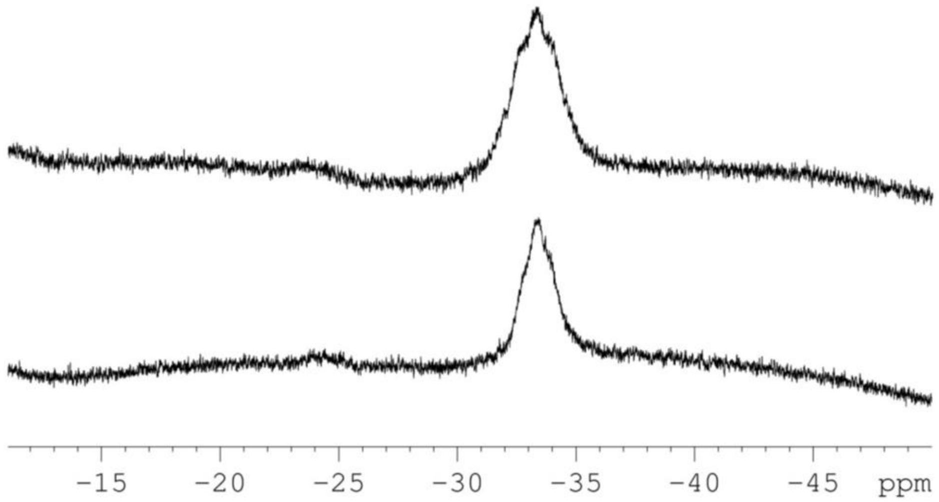

Figure S14: (*bottom*) $^{11}B\{^{1}H\}$ (128 MHz), (*top*) ^{11}B (128 MHz) NMR spectra of **3b** (from polymerization reaction in toluene, 100 ^{o}C, 18 h) in THF-d8.

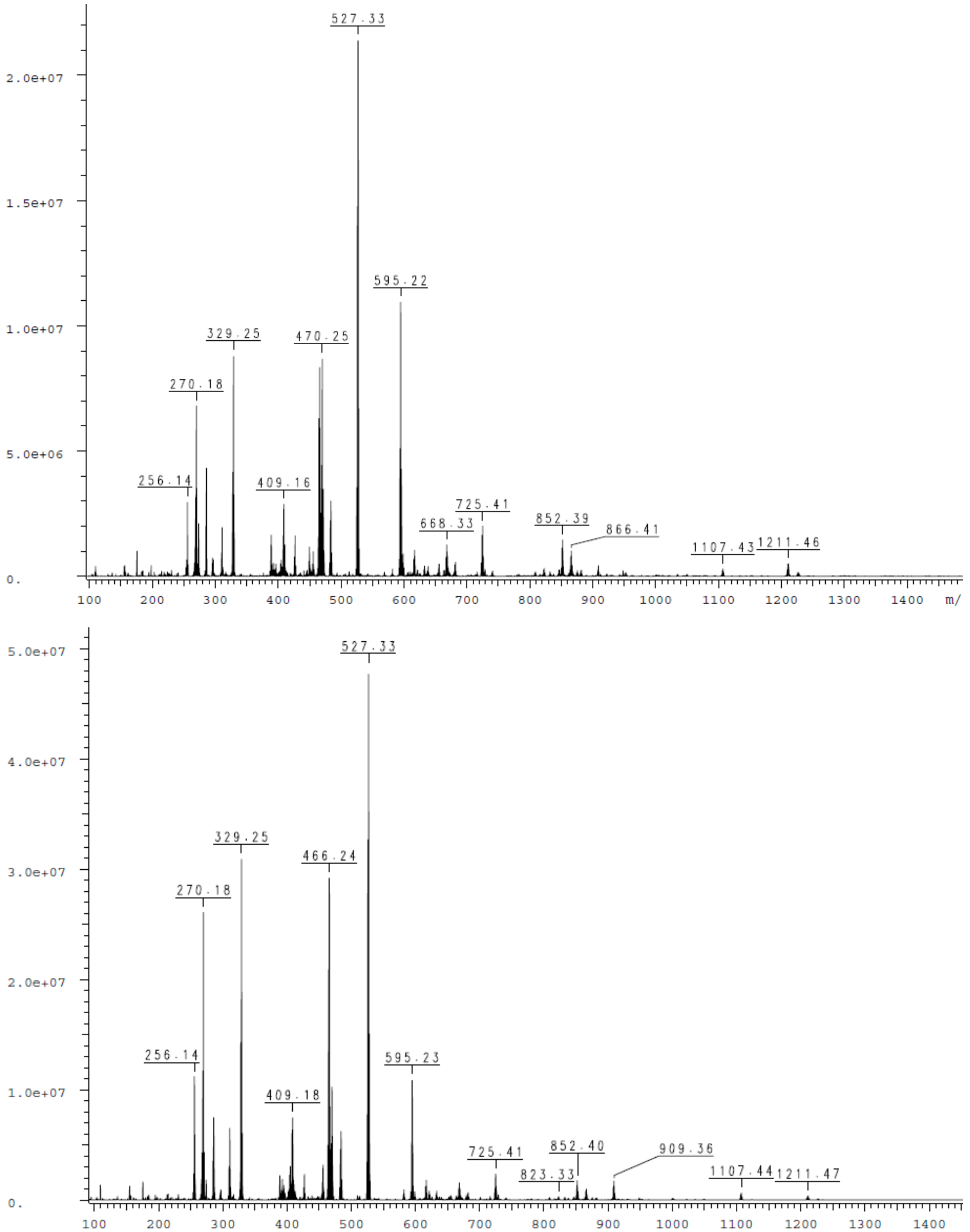

Figure S15: ESI – mass spectra of **3b** (from polymerization reaction in toluene, 100 °C, 18 h) in THF (*top*), and toluene (*bottom*).

Polyphosphinoborane **3b** was analysed by DLS. All samples were characterised by correlation function with a high *y*-intercept, which is indicative of the presence of particles within the measurable size range (1nm – 5μm). It proved impossible, however, to satisfactorily fit the decay of these functions, therefore suggesting that they result from the sum of multiple exponentials and the samples were ultimately too polydisperse for analysis.

Polymerization of ^{t}BuHP-BH$_2$·NMe$_3$ (1c) to form [^{t}BuHP-BH$_2$]$_n$ (3c)

Three methods were employed:

a) 100 mg (0.62 mmol) of ^{t}BuHP-BH$_2$·NMe$_3$ was heated at 40°C for 48 h.
b) 410 mg (2.55 mmol) of ^{t}BuHP-BH$_2$·NMe$_3$ was dissolved in 10 mL toluene and heated at 40°C for 48 h.
c) 500 mg (3.11 mmol) of ^{t}BuHP-BH$_2$·NMe$_3$ was dissolved in 10 mL toluene and stirred at room temperature (22 ^{o}C) for 48 h.

After removing all the volatiles under vacuum, the remaining polymer was dissolved in a minimum amount of toluene (*ca.* 1.5 mL). The viscous solution was added drop wise to vigorously stirred acetonitrile (*ca.* 200 mL) and a white rubber-like solid precipitated. The solid was filtered off and dried under vacuum.

Yield of (**3c**): a) 125 mg (40 %)
b) 120 mg (46 %)
c) 250 mg (80 %)

The NMR shifts and corresponding spectra from the polymerization reaction in toluene, 22 ^{o}C, 48 h (c), are shown below. The NMR spectra obtained from the 48 h polymerization reactions in the absence of solvent (a), and in toluene (b) at 40 ^{o}C are similar.

^{1}H NMR (400 MHz, CDCl$_3$): δ = 0.6 -1.6 (s, v br, BH$_2$), 1.18 (s, br, P^{t}Bu), 3.73 (d, br, $^{1}J_{H,P}$ = 330 Hz PH). ^{31}P NMR (162 MHz, CDCl$_3$): δ = –18 to –25 (br, PH). ^{31}P{^{1}H} NMR (162 MHz, CDCl$_3$): δ = –18 to –25 (br, PH). ^{11}B NMR (128 MHz, CDCl$_3$): δ = –38 (br, BH$_2$). ^{11}B{^{1}H} NMR (128 MHz, CDCl$_3$): δ = –38 (br, BH$_2$). ^{13}C NMR (101 MHz, CDCl$_3$) δ = 26.9 (s, ^{t}Bu), 27.5 – 29.2 (m, v br, ^{t}Bu).

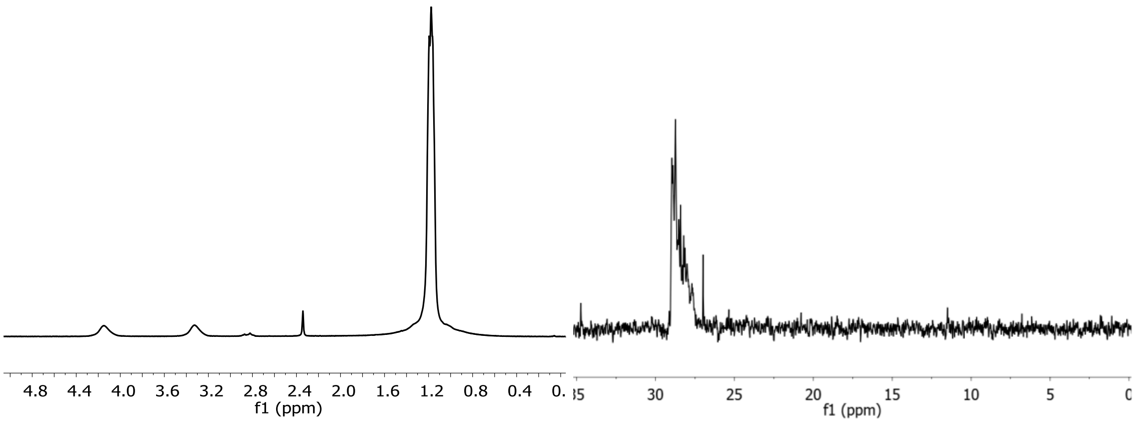

Figure S16: (*left*) ^{1}H (400 MHz), (*right*) ^{13}C (101 MHz) NMR spectra of **3c** (from polymerization reaction in toluene, 22 ^{o}C, 48 h) in CDCl$_3$.

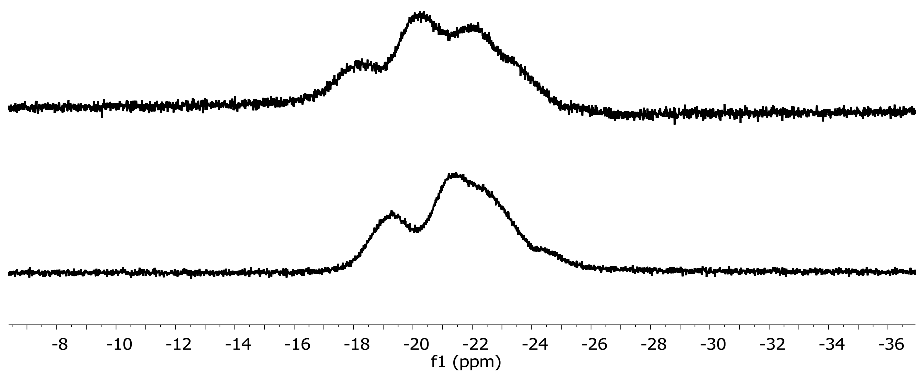

Figure S17: (*bottom*) $^{31}P\{^1H\}$ (121 MHz), (*top*) ^{31}P (121 MHz) NMR spectra of **3c** (from polymerization reaction in toluene, 22 ^{o}C, 48 h) in $CDCl_3$.

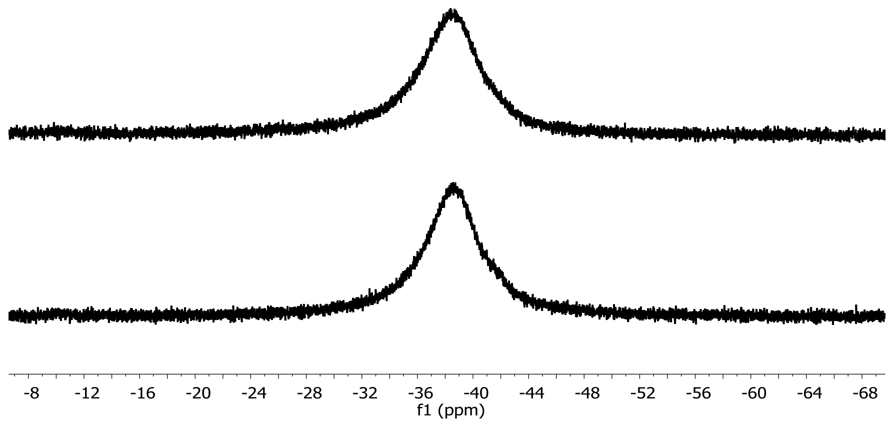

Figure S18: (*bottom*) $^{11}B\{^1H\}$ (96 MHz), (*top*) ^{11}B (96 MHz) NMR spectra of **3c** (from polymerization reaction in toluene, 22 ^{o}C, 48 h) in $CDCl_3$.

GPC analysis conducted with THF as eluent resulted in two peaks. A lower molecular weight component ranging from M_n = 17 000 to 22 400 g mol^{-1}, and an apparent higher molecular weight component ranging from M_n = 197 000 to 220 000 g mol^{-1} (Figure S21). The lower molecular weight component is consistent with DLS data (see manuscript, and Figure S20). The absence of a size population by DLS that corresponds to the high molecular weight component is suggestive that the presence of this species is dependent on the conditions applied, and most probably larger aggregates form in diluted THF solutions but not in solutions of CH_2Cl_2. Similar phenomena have been observed for poly(phenylphosphinoborane)[7], where the concentration of aggregates decreases with time and is effectively zero after stirring at room temperature for one day.

Repeated GPC analysis on samples stirred at room temperature for one day showed no significant difference to those analyzed immediately after dissolving in THF. This suggests that if aggregates are present then they are either kinetically more stable than those of the phenyl analogue, or THF is only a marginal solvent for the material (**3c**) prepared in this study.

GPC analysis was then conducted with $CHCl_3$ as eluent. The results are discussed in the main text. See Figures 3 and S22. The results were consistent with DLS data and indicated that these were true values for unimolecularly dissolved polymer chains in solution.

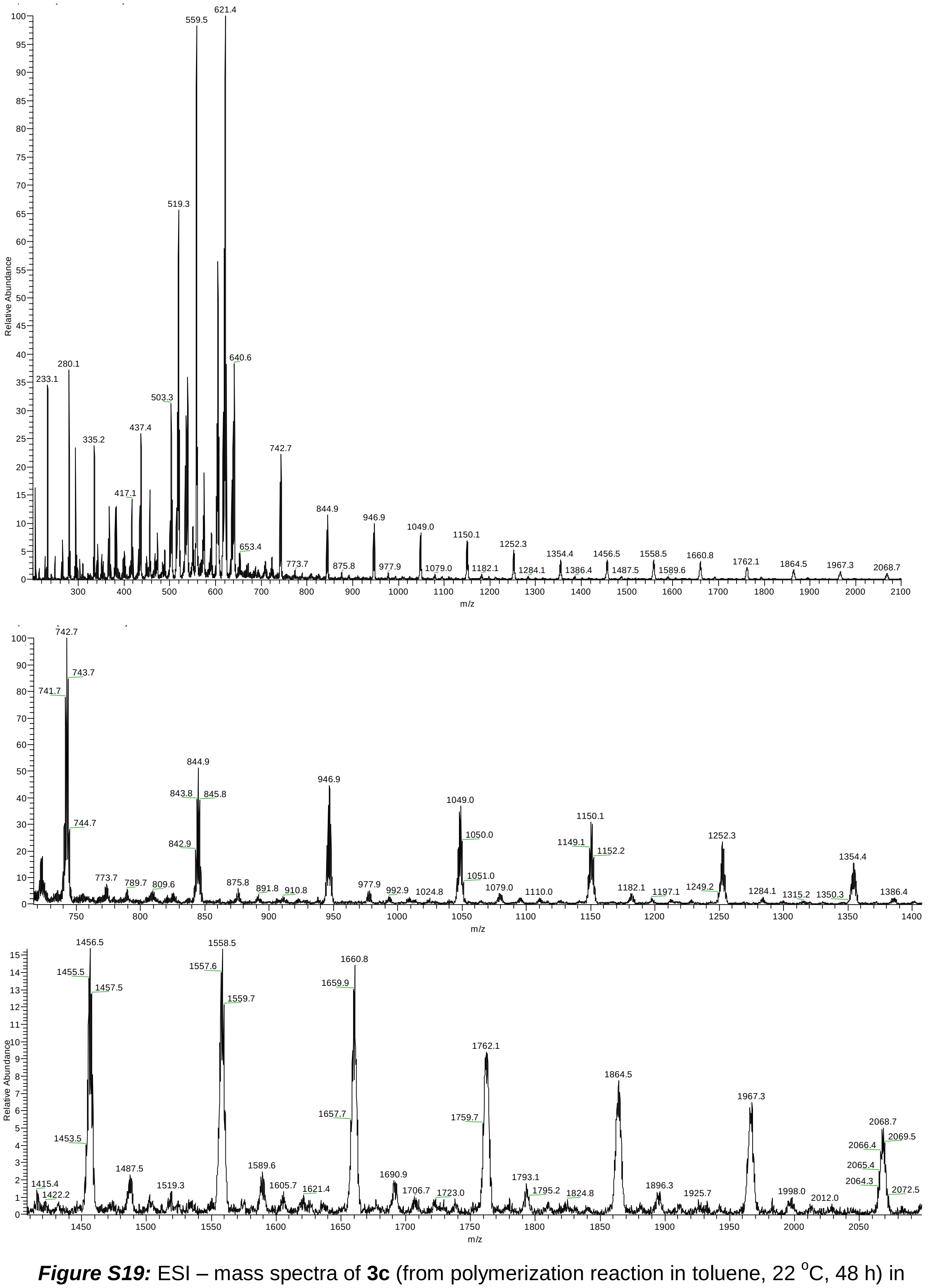

Figure S19: ESI – mass spectra of **3c** (from polymerization reaction in toluene, 22 ^{o}C, 48 h) in acetonitrile.

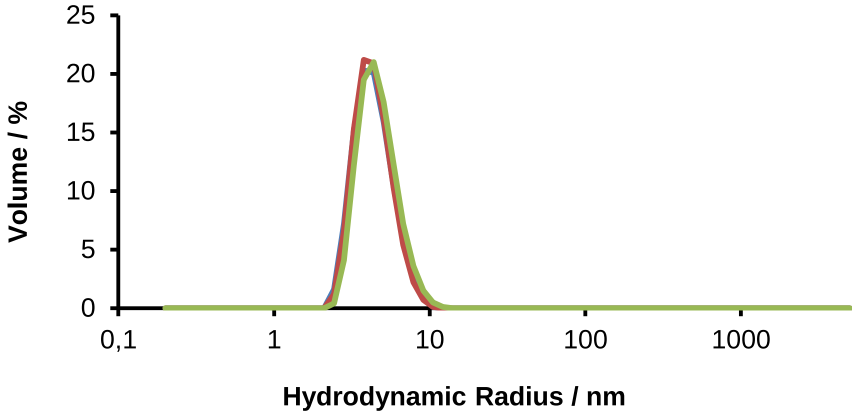

Figure S20: DLS chromatogram of **3c** in $CHCl_3$ isolated from: *blue* (neat, 40 ^oC, 48 h) R_h = 5.5 nm; *green* (in toluene, 40 ^oC, 48 h) R_h = 4.4 nm; *red* (in toluene, 22 ^oC, 48 h) R_h = 5.1 nm.

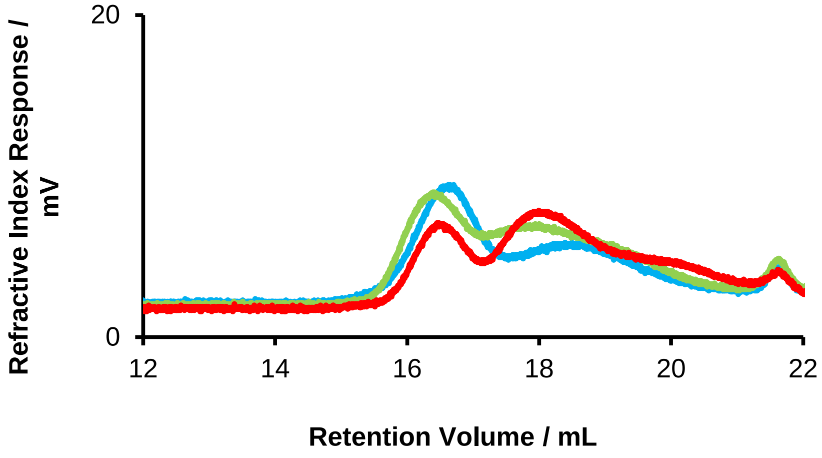

Figure S21: GPC chromatogram of **3c** in THF: *blue* (neat, 40 ^oC, 48 h); *green* (in toluene, 40 ^oC, 48 h); *red* (in toluene, 22 ^oC, 48 h).

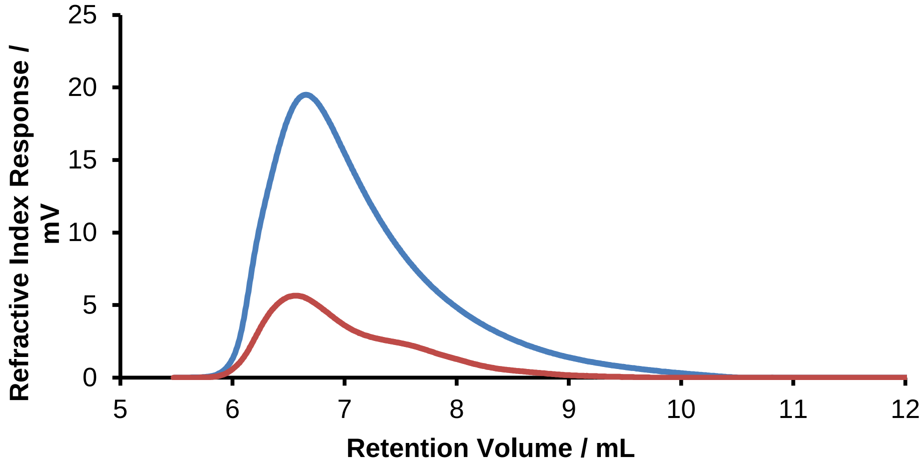

Figure S22: GPC chromatogram of **3c** in $CHCl_3$ isolated from: *red* (neat, 40 ^oC, 48 h) M_n = 27 800 g mol^{-1}, PDI = 1.9; *blue* (in toluene, 22 ^oC, 48 h) M_n = 35 000 g mol^{-1}, PDI = 1.6.

Catalytic dehydrocoupling of $^{t}BuPH_2{\cdot}BH_3$ by $Cp(CO)_2Fe(OSO_2CF_3)$ (5 mol%). To a 1.0 M solution of $^{t}BuPH_2{\cdot}BH_3$ (83 mg, 0.80 mmol), in 0.8 mL of anhydrous toluene was added 5 mol% of $Cp(CO)_2Fe(OSO_2CF_3)$ (12.8 mg, 0.04 mmol). The solution was then charged into a quartz J. Young NMR tube, which was subsequently sealed and allowed to react at 100 ^{o}C. The solution turned from dark red to bright yellow within the first hour, and remained so throughout the duration of the reaction. Progress was monitored *in situ* by $^{31}P\{^{1}H\}$ (121 MHz) and $^{11}B\{^{1}H\}$ (96 MHz) NMR spectroscopy for the formation of polyphosphinoborane (Figure S23). After consumption of $^{t}BuPH_2{\cdot}BH_3$ (176 h), the reaction solution was transferred into a beaker of cold pentane (-78 ^{o}C), which resulted in the precipitation of product. The solution was filtered and washed several times (3 x 2 mL) with cold pentane and dried under vacuum to afford an amber gum (38 mg, 47% yield). $^{31}P\{^{1}H\}/^{31}P$ (121 MHz) and $^{11}B\{^{1}H\}$ (96 MHz) NMR spectroscopy of isolated product in toluene are identical to *in situ* experiments and indicate the presence of multiple broad, ill-defined components ($\delta^{11}B$ -40 ppm, $\delta^{31}P$ -11 ppm).

Catalytic dehydrocoupling of $^{t}BuPH_2{\cdot}BH_3$ by $Cp(CO)_2Fe(OSO_2CF_3)$ (5 mol%), large scale. To a 1.0 M solution of $^{t}BuPH_2{\cdot}BH_3$ (400 mg, 3.80 mmol), in 3.8 mL of anhydrous toluene was added 5 mol% of $Cp(CO)_2Fe(OSO_2CF_3)$ (61 mg, 0.19 mmol). The solution was then charged into a J. Young reaction vessel, which was subsequently sealed and allowed to react at 100 ^{o}C for 176 h. The solution turned from dark red to bright yellow within the first hour, and remained so throughout the reaction. After 176 h, the reaction solution was then quickly transferred into a beaker of cold pentane (-78 ^{o}C), which resulted in the immediate precipitation of product. The solution was filtered and washed several times (3 x 2 mL) with cold pentane and dried under vacuum to afford an amber gum (134 mg, 34% yield). $^{31}P\{^{1}H\}/^{31}P$ (121 MHz) and $^{11}B\{^{1}H\}$ (96 MHz) NMR spectroscopy of the isolated compound in toluene are identical to *in situ* experiments (*vide supra*) and indicate the presence of multiple, ill-defined components (see Figures S23 and S24). ESI-MS revealed multiple components, with weights no higher than ~1100 g/mol, but with anticipated fragmentation patterns for loss of 102 m/z, corresponding to [$^{t}BuPH{\cdot}BH_2$] (Figure S25). GPC analysis of a 2 mg/mL $CHCl_3$ solution of the dark amber gum with $CHCl_3$ as eluent revealed no components within the calibrated range (i.e. greater than ca. 1000 g mol^{-1}). The absence of a peak corresponding to high molar mass material from GPC analysis, in conjuction with the results from ESI-MS indicated a polydisperse mixture of low molecular weight oligomers (~10 units or less as detected).

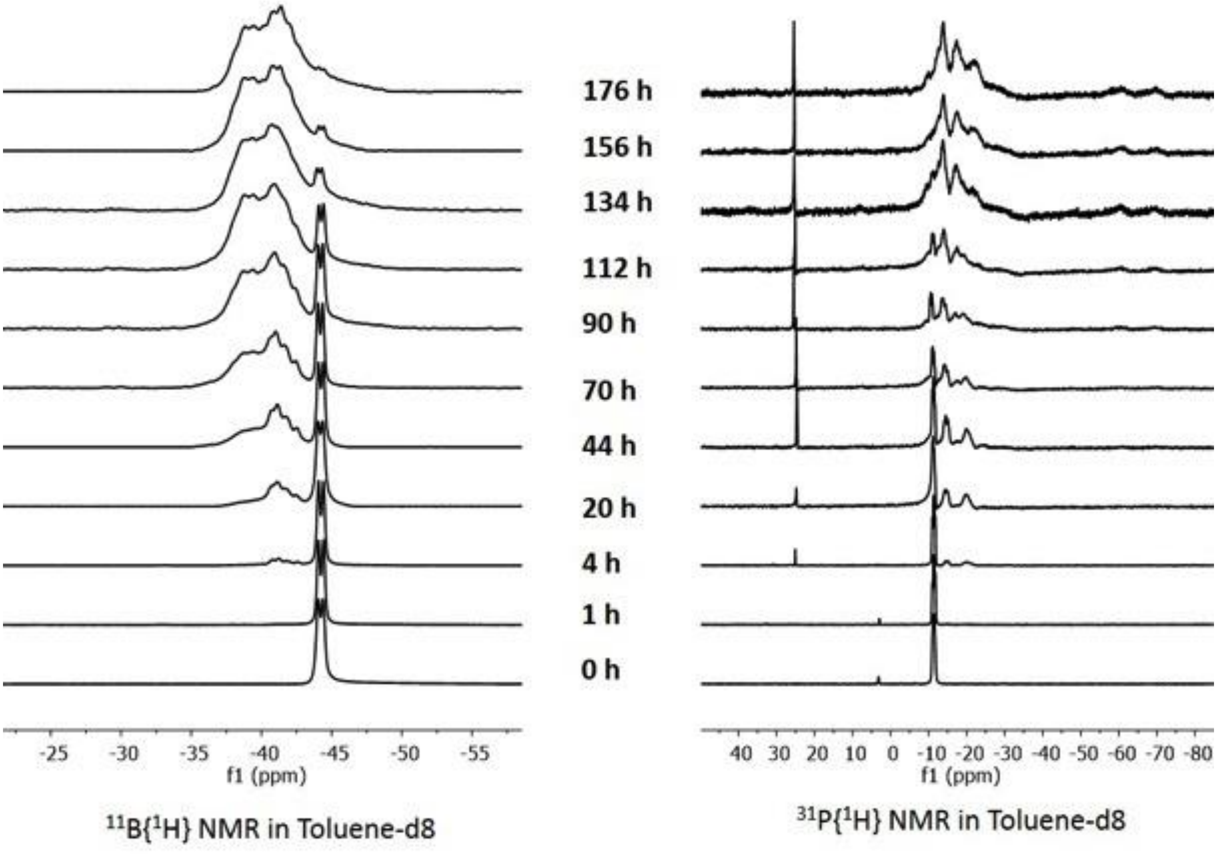

Figure S23: (*left*) $^{11}B\{^1H\}$ (96 MHz), (*right*) $^{31}P\{^1H\}$ (121 MHz) NMR reaction profiles for the dehydrocoupling reaction of $^tBuPH_2{\cdot}BH_3$ to oligomers of $[^tBuPH\text{-}BH_2]_n$ with 5 mol% $Cp(CO)_2Fe(OSO_2CF_3)$ as catalyst in toluene d-8 at 100 ^oC.

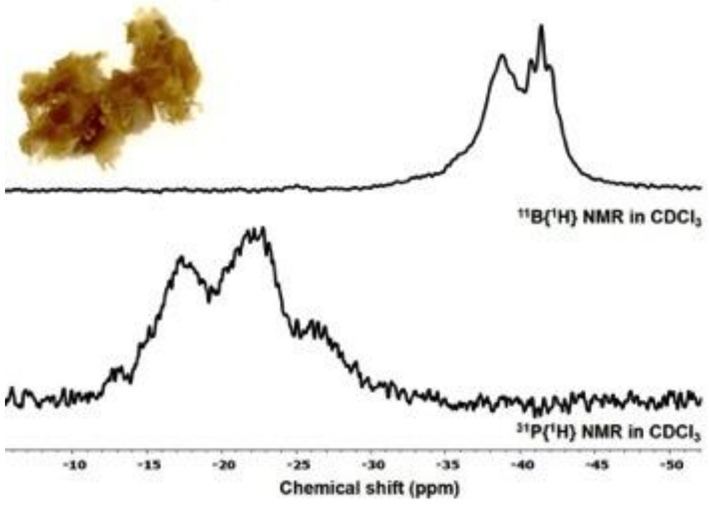

Figure S24: (*bottom*) $^{31}P\{^1H\}$ (121 MHz), (*top*) $^{11}B\{^1H\}$ (96 MHz) NMR spectra of $[^tBuPH\text{-}BH_2]_n$ oligomers in $CDCl_3$; isolated from the large scale reaction of $^tBuPH_2{\cdot}BH_3$ with 5 mol% $Cp(CO)_2Fe(OSO_2CF_3)$ as catalyst in toluene (100 ^oC, 176 h); (*inset*) photo of isolated oligomers.

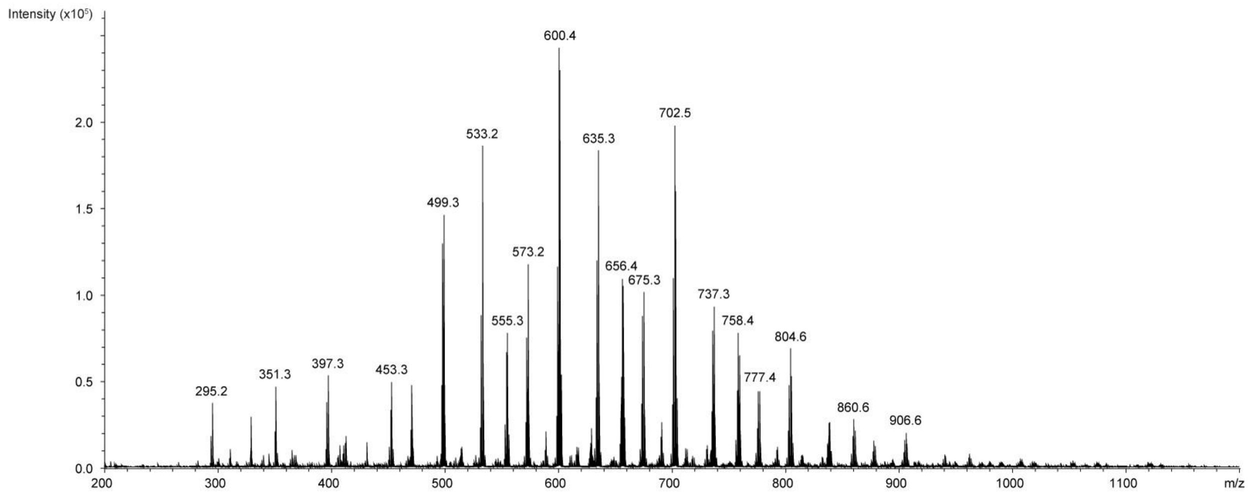

Figure S25: ESI – mass spectrum of [^{t}BuPH-BH$_2$]$_n$ oligomers in THF, isolated from the large scale reaction of ^{t}BuPH$_2$·BH$_3$ with 5 mol% Cp(CO)$_2$Fe(OSO$_2$CF$_3$) as catalyst in toluene (100 ^{o}C, 176 h).

Crystallographic details

Table S1. Crystallographic data for compounds 1b and 1c.

	**1b**	**1c**
Empirical formula	$C_{15}H_{21}BNP$	$C_7H_{21}BNP$
Formula weight *M*	257.11 g/mol	161.03 g/mol
Crystal	colourless block	colourless block
Crystal size [mm^3]	0.15 x 0.08 x 0.07	0.38 x 0.14 x 0.06
Temperature *T*	123(1) K	123(1) K
Crystal system	orthorhombic	orthorhombic
Space group	$Pca2_1$	*Pnma*
Unit cell dimensions	*a* = 16.5779(2) Å	*a* = 10.1552(2) Å
	b = 6.2048(1) Å	*b* = 10.1654(3) Å
	c = 14.2632(1) Å	*c* = 11.0614(2) Å
	α = 90°	*α* = 90°
	β = 90°	*β* = 90°
	γ = 90°	*γ* = 90°
Volume *V*	1467.15(3) $Å^3$	1141.89(5) $Å^3$
Formula units *Z*	4	4
Absorption coefficient $\mu_{Cu\text{-}K\alpha}$	1.488 mm^{-1}	1.660 mm^{-1}
Density (calculated) ρ_{calc}	1.164 g/cm^3	0.937 g/cm^3
F(000)	552	360
Theta range $\theta_{min}/\theta_{max}$	5.336 / 66.511°	5.912 / 67.044°
Absorption correction	analytical	multi-scan
Index ranges	$-18 < h < 19$	$-11 < h < 12$
	$-5 < k < 7$	$-13 < k < 12$
	$-16 < l < 16$	$-13 < l < 13$
Reflections collected	6445	8996
Independent reflections [$I > 2\sigma(I)$]	2317 (R_{int} = 0.0236)	831 (R_{int} = 0.0480)
Completeness to full θ	0.970	0.988
Transmission T_{min} / T_{max}	0.860 / 0.925	0.848 / 1.000
Data / restraints / parameters	2364 / 1 / 214	1074 / 18 / 110
Goodness-of-fit on F^2 *S*	1.031	1.014
Final *R*-values [$I > 2\sigma(I)$]	R_1 = 0.0261	R_1 = 0.0344
	wR_2 = 0.0695	wR_2 = 0.1022
Final *R*-values (all data)	R_1 = 0.0268	R_1 = 0.0453
	wR_2 = 0.0706	wR_2 = 0.1087
Largest difference hole	–0.140	–0.117
and peak $\Delta\rho$	0.233 $eÅ^{-3}$	0.208 $eÅ^{-3}$
Flack parameter	0.007(9)	-

All crystal manipulations were performed under mineral oil or perfluorinated oil. The diffraction experiments were performed at 123 K on an Agilent Technologies Gemini R Ultra diffractometer or an Agilent SuperNova diffractometer with Cu-K_α or Mo- K_α radiation. Crystallographic data together with the details of the experiments are given in Table 1. The cell determination, data reduction and absorption correction for all compounds were performed with the help of the CrysAlis PRO software by Agilent Technologies Ltd. The full-matrix least-square refinement against F^2 was done with ShelXL. During the refinement several restraints and constraints had to be applied. For the description of the refinement strategy we list the commonly used syntax for the ShelXL program (DFIX, SADI, SIMU, ISOR, EADP). All atoms except hydrogen were refined anisotropically if not described otherwise. The H atoms were calculated geometrically and a riding model was used during refinement process. Graphical material was created with the free software Olex2. CCDC-1415883 (**1b**) and CCDC-1415884 (**1c**) contain the supplementary crystallographic data for this paper. These data can be obtained free of charge from The Cambridge Crystallographic Data Centre via www.ccdc.cam.ac.uk/data_request/cif.

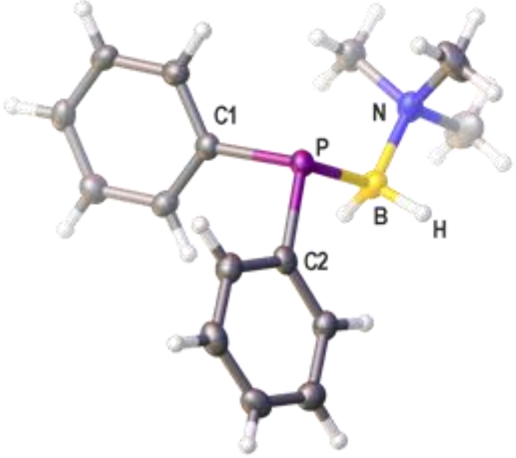

Figure S26. Molecular structure of compound **1b** determined by single crystal X-Ray structure analysis (thermal ellipsoids drawn at 50% probably level). Selected bond lengths [Å] and angles[°]: P–B 1.975(2), P-C1 1.849(2), P-C2 1.847(2), B-N 1.619(3), C1–P–C2 99.3(8), C1–P–B 105.7(1), C2–P–B 102.1(1), P–B–N 112.4(2).

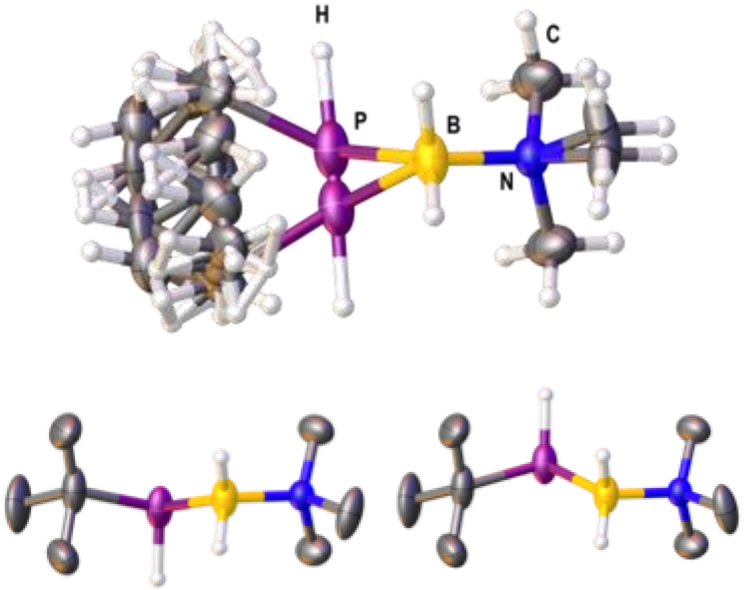

Figure S27. Molecular structure of compound **1c** determined by single crystal X-Ray structure analysis (thermal ellipsoids drawn at 50% probably level). (*top*) heavily disordered structure, (*bottom*) the two enantiomers present. Selected bond lengths [Å] and angles [°]: P–B 1.985(2), P–C 1.890(2), N–B 1.621(2), B–P–C 102.7(1), P–B–N 108.9(1).

References

[1] C. Marquardt, C. Thoms, A. Stauber, G. Balazs, M. Bodensteiner, M. Scheer, *Angew. Chem. Int. Ed.* **2014**, *53*, 3727 – 3730.

[2] C. Marquardt, A. Adolf, A. Stauber, M. Bodensteiner, A. V. Virovets, A. Y. Timoshkin, M. Scheer, *Chem. Eur. J.* **2013***, 19*, 11887 – 11891.

[3] W. A. Herrmann, G. Brauer, *Synthetic Methods of Organometallic and Inorganic Chemistry*, *Vol.* 3 *,* **1996** *,* Thieme Publishers*,* Stuttgart.

[4] Agilent Technologies **2006-2011**, CrysAlisPro Software system, different versions, Agilent Technologies UK Ltd, Oxford, UK.

[5] A. Altomare, M. C.Burla, M. Camalli, G. L. Cascarano, C. Giacovazzo, A. Guagliardi, A.G. G. Moliterni, G. Polidori, R. Spagna, *J. Appl. Cryst.* **1999**, *32*, 115-119.

[6] G. M. Sheldrick, *Acta Cryst.* **2008**, *A64*, 112–122.

[7] O.V. Dolomanov, L.J. Bourhis, R.J. Gildea, J.A.K. Howard, H. Puschmann, OLEX2: A complete structure solution, refinement and analysis program, *J. Appl. Cryst*., **2009**. *42*, 339-341.

[7] H. Dorn, R. A. Singh, J. A. Massey, J. M. Nelson, C. A. Jaska, A. J. Lough, I. Manners, *J. Am. Chem. Soc.* **2000**, *122*, 6669–6678.
